# Non-targeted Screening of Natural Products from 288 Fungal Endophytes from Canadian Fruit Crops

**DOI:** 10.1021/acsomega.3c02786

**Published:** 2023-06-27

**Authors:** Natasha DesRochers, Justin B. Renaud, Joey B. Tanney, Ashraf Ibrahim, Ken K.-C. Yeung, Mark W. Sumarah

**Affiliations:** † London Research and Development Centre, Agriculture and Agri-Food Canada, 1391 Sandford Street, London, Ontario N5V 4T3, Canada; ‡ Department of Chemistry, University of Western Ontario, 1151 Richmond Street, London, Ontario N6A 3K7, Canada; § Pacific Forestry Centre, Canadian Forest Service, Natural Resources Canada, Victoria, British Columbia V8Z 1M5, Canada; ∥ McMaster University, 1290 Main St. W, Hamilton, Ontario L8S 4L8, Canada; ⊥ Department of Biochemistry, University of Western Ontario, 1151 Richmond Street, London, Ontario N6A 3K7, Canada

## Abstract

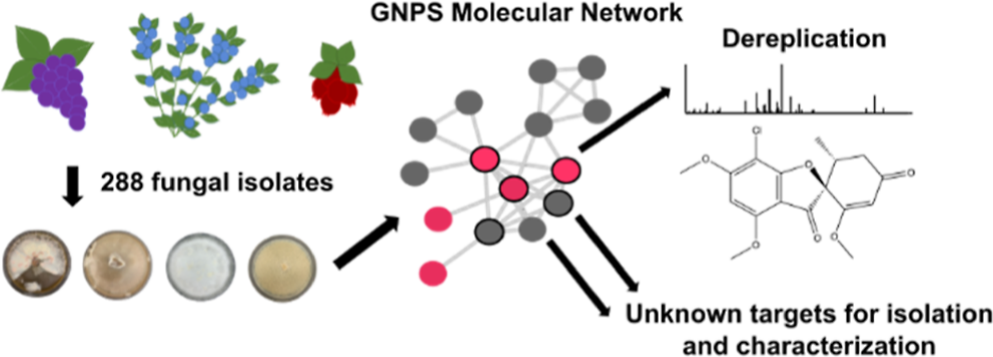

Many diverse species of fungi naturally occur as endophytes in plants. The majority of these fungi produce secondary metabolites of diverse structures and biological activities. Culture extracts from 288 fungi isolated from surface-sterilized blueberries, cranberries, raspberries, and grapes were analyzed by LC–HRMS/MS. Global Natural Products Social (GNPS) Molecular Networking modeling was used to investigate the secondary metabolites in the extracts. This technique increased the speed and simplicity of dereplicating the extracts, targeting new compounds that are structurally related. In total, 60 known compounds were dereplicated from this collection and seven new compounds were identified. These previously unknown compounds are targets for purification, characterization, and bioactivity testing in future studies. The fungal endophytes characterized in this study are potential candidates for providing bio-protection to the host plant with a reduced reliance on chemical pesticides.

## Introduction

Fungi that live in plant tissue without causing disease are called “endophytes”. Many plants from grasses to conifers to some seaweeds have formed mutualisms with fungi. In some cases, the benefit to the plant is clear in terms of the fungal metabolites reducing herbivory or fungal disease.^1^ Natural products from fungal endophytes are of particular interest to study as they exhibit a wide range of bioactivities. Some endophyte metabolites have been used in medical applications in their pure form, such as topical emodepside, for the treatment of nematode infections in cats.^2,3^ They may also be used to inoculate cool season fescues or white spruce with their respective endophytes that produce anti-insectan compounds that deter harmful pests.^4^

To better understand the endophyte species diversity that exists in Canadian fruit crops, we isolated fungal endophytes from highbush and lowbush blueberries (248 isolates from *Vaccinium angustifolium* and *Vaccinium corymbosum*), grapes (14 isolates from *Vitis vinifera*), cranberries (18 isolates from *Vaccinium macrocarpon*), pear (1 isolate from *Pyrus communis*), and raspberries (7 isolates from *Rubus idaeus*). Between 2011 and 2015, nearly 300 strains of fungi were isolated from the leaves and stems of these plant samples and were identified at the species level, where possible. Building on this work, there is a need to explore the natural products produced by these strains to better understand their role in the host-endophyte relationship.

To date, our preliminary investigations within this collection resulted in the discovery of several novel and bioactive secondary metabolites including the antifungal polyketides trienylfuranones from the raspberry endophyte *Hypomontagnella submonticulosa*,^5^ the antibacterial non-ribosomal peptides ellisiiamides from the blueberry-*Pinus* endophyte *Xylaria ellisii*,^6,7^ and the antimicrobial polyketides nemanilactones and nemanifuranones from the grape endophyte tentatively identified as *Nemania serpens*.^8^ However, only a fraction of the species within the collection have been investigated for their ability to produce novel compounds. In a classical natural product discovery approach, fungal isolates are grown in a variety of culture conditions. When there is sufficient growth, the metabolites are extracted, purified, and characterized when possible. This process is laborious and, in the aforementioned discoveries, further complicated by the typically slow growth rate of endophyte species.

Another aspect of the classical discovery approach is to screen crude extracts in one or more bioassays and identify the compounds responsible for any assay hits. However, these approaches often lead to the identification of the most abundant bioactive compounds, which are generally known compounds. More importantly this can miss minor novel compounds. A metabolomics-guided approach may also be taken, where culture extracts are first screened by LC–HRMS and are grouped statistically using multivariate analysis, such as orthogonal projections to latent structures discriminant analysis (OPLS-DA) or principal component analysis (PCA), based on their secondary metabolite profiles.^6^ This approach allows strains with common metabolites to be grouped together, while unique metabolite profiles can be readily identified as divergent outliers. Furthermore, the datasets can be rapidly dereplicated or, in other words, have known compounds identified, saving time and costly efforts in purification and structural characterization.^9^

The complex biosynthetic pathways that create natural products often result in the existence of chemical classes, rather than unique individual compounds.^10^ Non-ribosomal peptides and polyketides are synthesized by large proteins with modular domains; each module executes a step in growing the chemical structure, therefore variation can occur if a module has relaxed specificity for its function.^10^ For example, the tyrocidines are a large class of cyclic decapeptides with antibiotic activity arising from three peptide synthetases. Tyrocidines exhibit structural variance due to the reduced specificity of these synthetases for aromatic amino acids at select locations along the peptide chain.^11^ This results in at least 28 tyrocidines with a common core sequence of amino acids that differ by the interchange of select aromatic amino acids.^12^ A similar biosynthetic mechanism is demonstrated by the prochlorosins—a group of cyclic, lanthionine-containing antimicrobial peptides derived from marine cyanobacteria. The nearly thirty prochlorosins are formed by the activity of one enzyme with exceptionally low substrate specificity.^13^

Regardless of compound class, tandem mass spectrometry (MS/MS) is a critical tool for natural product discovery. The MS/MS fragmentation patterns of natural products are highly dependent on their molecular structure, meaning structurally similar compounds often follow common fragmentation pathways leading to diagnostic product ions and/or neutral loss products. For some compound classes, dereplication methods are advanced, such as for peptidic natural products via iSNAP—informatic search algorithm for natural products—which dereplicates non-ribosomal peptides against a database of in-silico tandem MS spectra.^14^ For other compound classes, the identification of structurally related unknowns can be a challenge, especially for complex classes such as polyketides. In recent years molecular networking has become a popular tool for addressing this gap in rapid compound identification.^15^ It takes advantage of the fragmentation similarities among compounds within a class to map chemicals visually according to their degree of relatedness. Each compound is represented by a node, connected to one another by lines called edges, if they share similar fragmentation sequences or transitions within their tandem MS spectra. The Global Natural Products Social (GNPS) Molecular Network takes this a step further with the ability to also compare compounds to a large publicly shared database of known fragmentation spectra and to other user-generated spectra for community identifications.

Unlike the OPLS-DA and PCA statistical approaches previously employed on our collection of endophyte extracts, which grouped strains based on metabolite correlations and abundance, GNPS groups the metabolites themselves according to their MS/MS spectral similarity. This additional information about structural relatedness can guide us toward unknown compounds that are related to known bioactive compounds or new chemotypes, giving us a more streamlined approach to prioritize the discovery of novel natural products.

In this work, we examine a collection of 288 fungal endophyte extracts using non-targeted analysis by LC–HRMS/MS and the GNPS molecular networking platform to explore the chemical diversity of fungi endogenous to agriculturally important fruiting plants to target novel natural products for further isolation and characterization. This approach has led to a more comprehensive understanding of endophytic fungal diversity seen across these plants and of the varied chemical profiles present among fungal species. It has also allowed for a focused effort to identify unique fungal isolates that produce novel compounds, while also identifying natural product targets with potential utility that warrant purification, characterization, and bioactivity testing.

## Results and Discussion

Based on ITS sequence similarity, the 288 endophyte strains represented 87 unique species across 71 genera. There are caveats with using only ITS to identify taxa, for example, it provides insufficient resolution for delineating species in common genera, such as *Aspergillus*, *Cladosporium*, and *Fusarium*, and species complexes comprising distinct species with identical or very similar ITS sequences.^16^ Furthermore, alpha diversity may be overestimated due to high intragenomic rDNA variation or conversely underestimated due to low or absent interspecific ITS variation.^17^ The species identifications provided here are considered tentative or first diagnoses pending sequencing with additional secondary barcodes.

**Figure 1:**
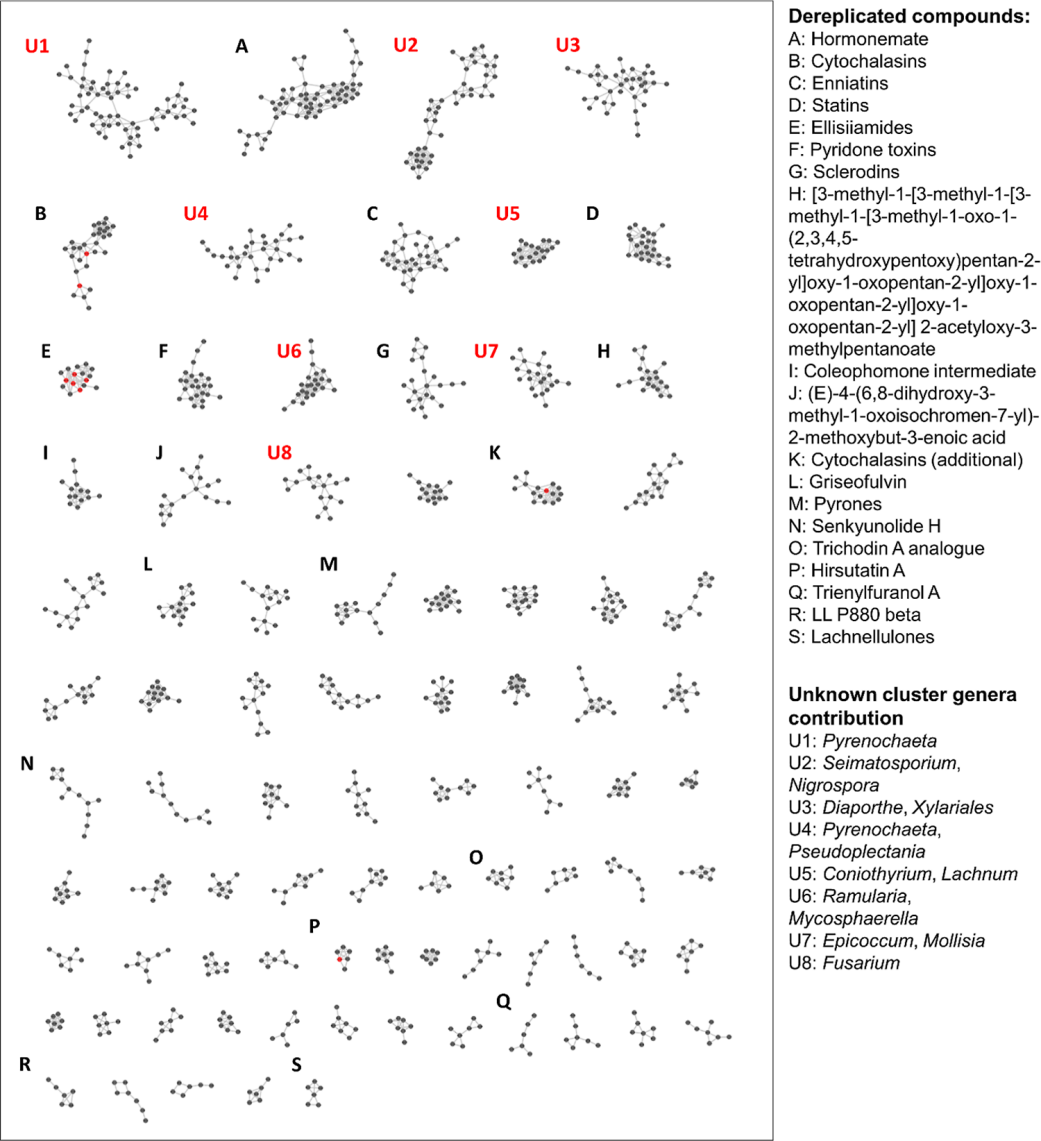
Molecular network of LC-HRMS/MS features from methanol extracts of Canadian fungal endophytes, generated with GNPS. Nodes indicate LC–MS/MS features, while lines indicate that the features share a cosine similarity score above the cutoff of 0.75. Red nodes indicate compounds from seed spectra, while gray nodes indicate compounds arising from endophyte extracts.

Endophyte extracts were analyzed by LC–MS/MS and data were subjected to molecular networking and dereplication using GNPS. Samples were dereplicated by comparison with the suite of libraries found within the GNPS search function, which include nearly 600,000 entries ranging from plant and microbial natural products, to human metabolites, to known drugs, and other reference standards. MS/MS spectra of the unknown samples were compared to the databases and were considered a match if their cosine score is above the user-assigned cut-off value of 0.75 for this study. Clusters that contain at least one known compound are labeled alphabetically in Figure 1. Unknown compounds within the dereplicated clusters are putatively structurally similar to the known compounds; their proposed chemical formulas are listed in Table S3. A subset of dereplicated clusters is examined in detail below, and the full list of dereplicated compounds is detailed in Table S2.

### PCA and GNPS Molecular Network

The performance of the LC–HRMS/MS system was evaluated by injecting quality control (QC) samples to assess instrument drift over the course of analysis. QC samples were made from pooled sample aliquots and were injected once every 10 test sample injections. Test and QC samples were subjected to PCA. While test samples are spread across the entire plot over a range of PC scores—as we would expect from a large group of diverse extracts—the QC samples group closely together due to their similar PC scores, indicating little variation among samples and, therefore, minimal instrumental drift (Figure S1).^18^ After assessing the reproducibility of the LC–HRMS/MS analysis, molecular networking with GNPS was performed. Seed spectra of known compounds were included in the network and belonged to compounds previously isolated from our fungal endophyte collection, including *Coniochaeta tetraspora*, *Nigrospora sphaerica*, *Sphaerulina vaccinii*, *Xylaria castorea*, and *X. ellisii*.^6,19^ All but *X. ellisii* were previously identified tentatively based on ITS data. Processing the LC–MS data by XCMS yielded ∼8000 molecular features in the PCA plot, therefore in the resulting molecular network, the cosine score cutoff was set at 0.75—slightly higher than the default setting of 0.6—to reduce the number of connections that warrant investigation. Following the removal of single-node clusters and features detected in the blank media controls, the molecular network contained 2804 nodes with 4119 connections (most of the resulting network is shown in Figure 1, with smaller clusters after S omitted to provide higher image resolution).

**Figure 2:**
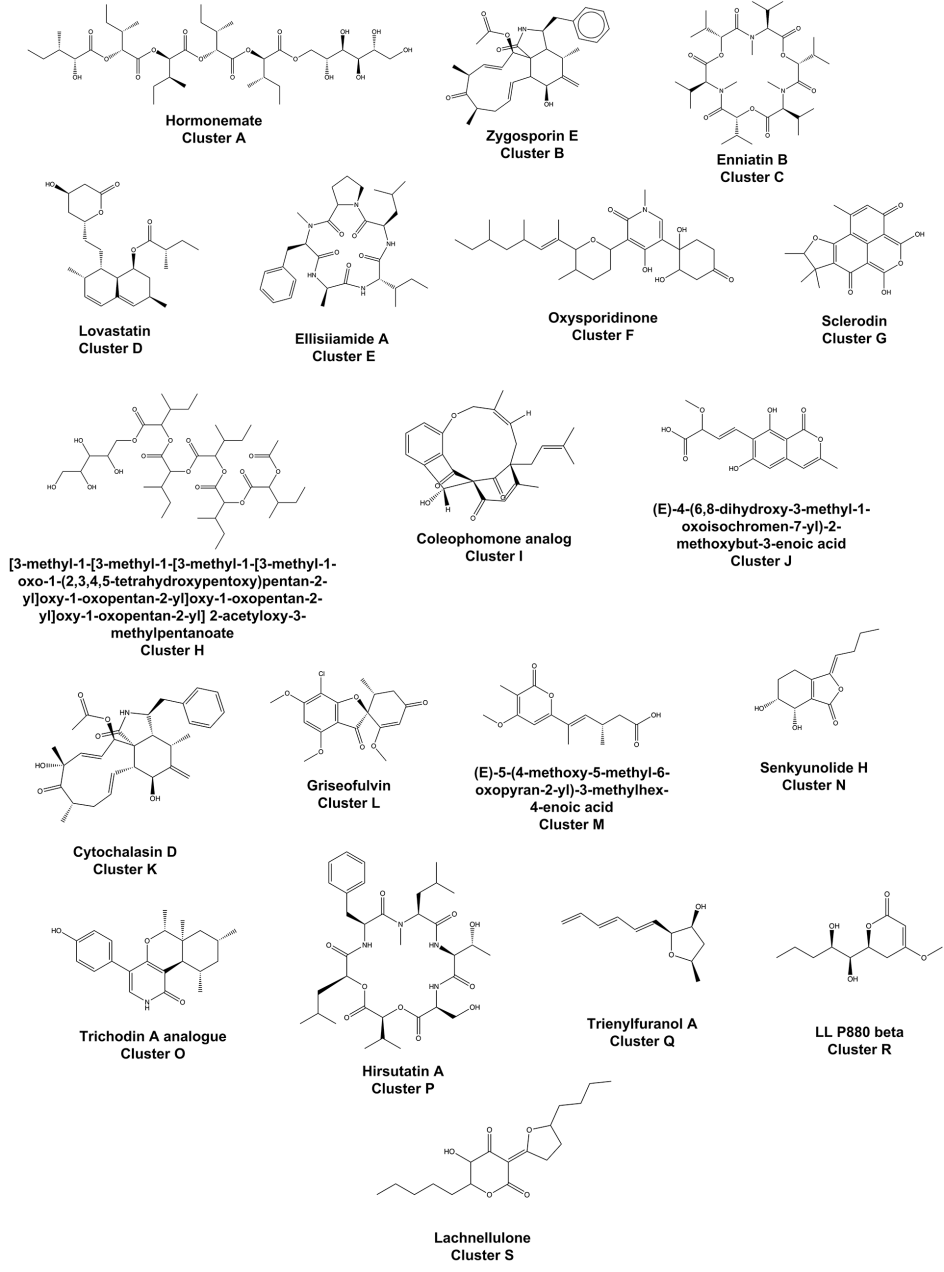
Representative structures for dereplicated compounds in the molecular network shown in Figure 1.

**Figure 3:**
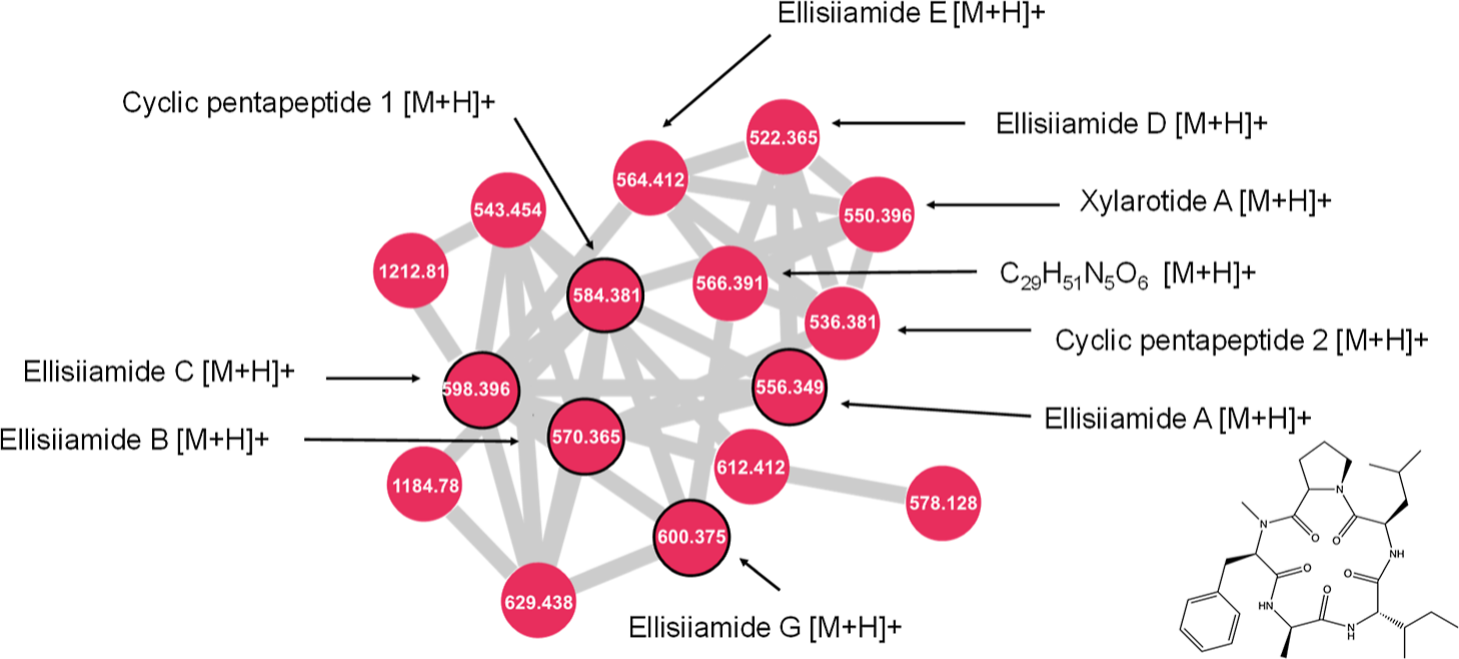
Close-up of cluster E from Figure 1. Ellisiiamides and related compounds are labeled. Nodes outlined in black were included as seed spectra. All nodes were detected in extracts of *X. ellisii*.

**Figure 4:**
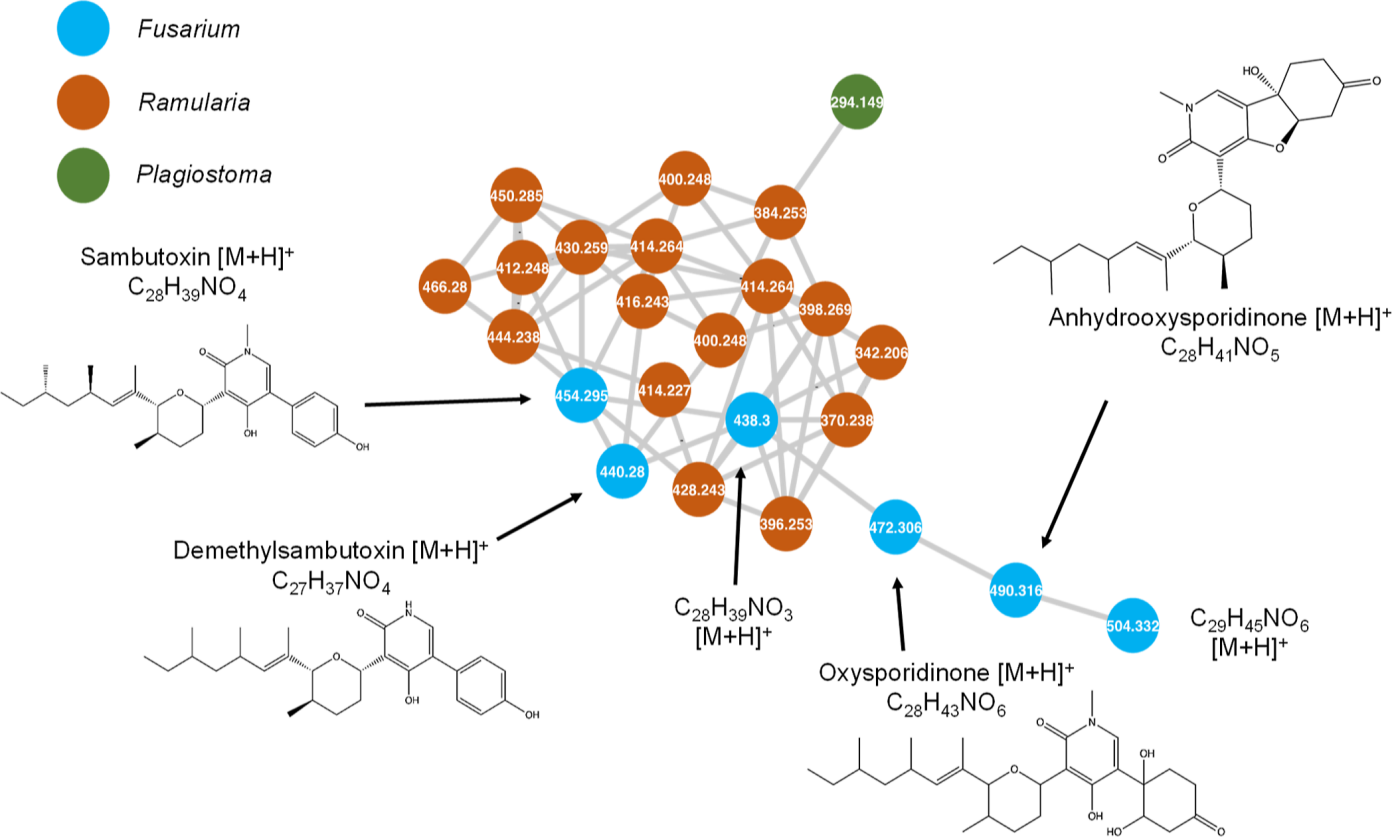
Close-up of cluster F from Figure 1. Oxysporidinone and related compounds are labeled. Blue nodes were produced exclusively by *Fusarium*.

**Figure 5:**
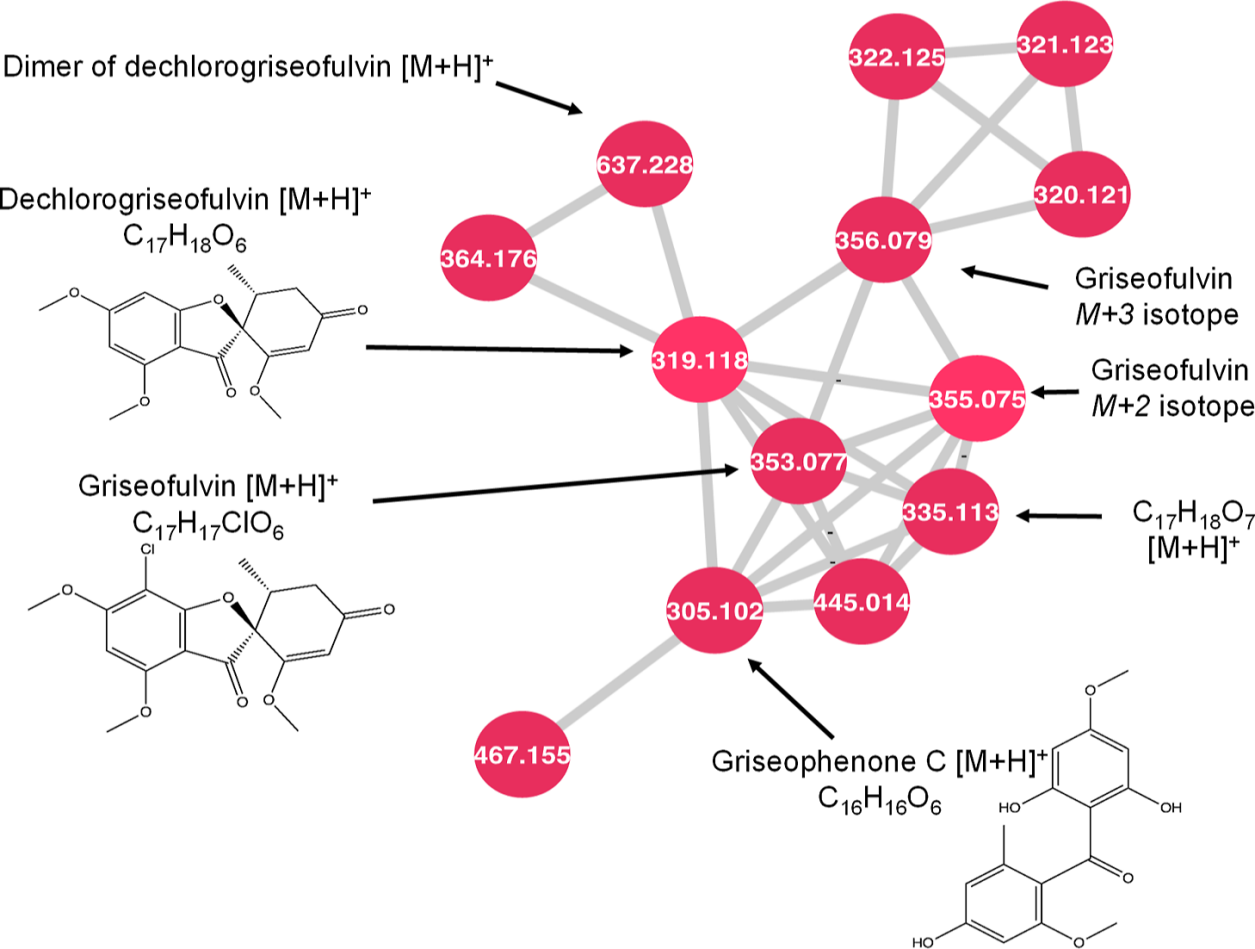
Close-up of cluster L from Figure 1. Griseofulvin and related compounds are labeled. All nodes were detected in multiple isolates of *X. ellisii*.

Sixty known compounds were dereplicated across 19 clusters in the network by comparison to the databases available through GNPS and to an in-house library of MS/MS spectra. These compounds are outlined in Figure 1. Dereplicated compounds listed in Supporting Information Table S2 cover a broad range of structures (Figure 2). We designated clusters containing one or more dereplicated compounds as “dereplicated clusters” (Figure 1; Table S3). Clusters that contain no dereplicated compounds represent the highest probability of being novel and are, therefore, ideal targets for future purification and characterization efforts. We classified these clusters as “unknown clusters” (Figure 1; Table S3). Of note, within the network, the positioning of clusters in relation to one another does not convey information, they are simply organized from largest to smallest (Figures 3–5).

### Cluster E—Ellisiiamides

Ellisiiamides A, B, C, and G, along with cyclic pentapeptide 1, were included as seed spectra in the network and, as expected, were identified among extracts of *X. ellisii*, the organism from which they were first identified.^6^ These grouped together in cluster E, along with ellisiiamide D, cyclic pentapeptide 2, and an unidentified congener with an *m*/*z* value of 566.3914 [M + H]^+^ and chemical formula of C_29_H_51_N_5_O_6_, tentatively identified as a new cyclic pentapeptide. It has a mass difference of 15.995 amu from xylarotide A, representing the addition of an oxygen atom and does not share a formula with any previously published members of this class. It also has a distinct retention time from xylarotide A, indicating that it is not an in-source fragment of xylarotide A. It is produced by two isolates of *X. ellisii* from the collection, namely E-206 and E-244. This new cyclic pentapeptide also shares high cosine similarity scores ranging from 0.86 to 0.93 with four dereplicated cyclic pentapeptides, lending further confidence to the identification.

Ellisiiamides A–C have previously been tested for bioactivity against *Candida albicans*, *Escherichia coli*, and *Saccharomyces cerevisiae*. Ellisiiamide A has modest activity against *E. coli* with an MIC of 100 μg/mL, while ellisiiamides B and C did not show any activity against the organisms tested. The remaining ellisiiamides have yet to be tested in bioassays, along with cyclic pentapeptide 1. Xylarotide A has been tested against *Bacillus pumilus*, *C. albicans*, *E. coli*, and *Staphylococcus aureus* and does not exhibit activity against any of them. However, other closely related cyclic pentapeptides from *Xylaria* spp. do exhibit bioactivity, such as cyclo(*N*-methyl-l-Phe-l-Val-d-Ile-l-Leu-l-Pro) from an endolichenic species of *Xylaria*, which shows synergistic antifungal activity with ketoconazole against *C. albicans*.^20^ It differs from ellisiiamide A only by a substitution of the alanine residue for a valine, and was only tested against *C. albicans*, indicating that more ellisiiamides may have additional bioactivities if tested against a broader range of organisms.

### Cluster F—Oxysporidinone

Oxysporidinone is an antifungal compound originally identified from the fungus *Fusarium oxysporum* and, here, was detected in extracts of *Fusarium* cf. *tricinctum*.^21^ It is cytotoxic to several plant pathogenic fungi, including *Alternaria alternata*, *Aspergillus niger*, *Botrytis cinerea*, and *Venturia inequalis*.^21^ Oxysporidinone is present within cluster F as a protonated ion (*m*/*z* 490.3162, [M + H]^+^) and was dereplicated with the GNPS library search. Several compounds within the cluster have chemical formulas similar to that of oxysporidinone, including formulas matching those of several known structural relatives of oxysporidinone. Sambutoxin was observed in *F.* cf. *tricinctum* extracts with an *m*/*z* value of 454.2952 [M + H]^+^. It is a mycotoxin produced by *Fusarium* spp. and has demonstrated toxicity in rats. The derivatives demethylsambutoxin and anhydrooxysporidinone were also present with *m*/*z* values of 440.2793 [M + H]^+^ and 472.3059 [M + H]^+^, respectively. Both are known products of *Fusarium* spp., but neither have recorded bioactivity with the small range of organisms they have been tested against. All dereplicated compounds were produced by all three isolates of *F.* cf. *tricinctum* in the collection.

Cluster F also contains two nodes representing chemical formulas of C_28_H_39_NO_3_ and C_29_H_45_NO_6_, given by *m*/*z* values of 438.3001 [M + H]^+^ and 504.3321 [M + H]^+^, respectively. The compound with the molecular formula C_28_H_39_NO_3_ resembles sambutoxin, with one less oxygen atom, and connected to sambutoxin by a high cosine similarity score of 0.98, indicating nearly identical fragmentation patterns. The formula of compound C_29_H_45_NO is one CH_2_ group larger than oxysporidinone, and the two are connected by a cosine similarity score of 0.85. These unidentified formulas did not match any published structural relatives within this compound class and are likely oxysporidinone relatives due to their high spectral similarity to known pyridine alkaloids. Both unknown compounds were produced by only two isolates of *F.* cf. *tricinctum*, namely E-178 and E-259. Other known structural relatives produced by *Fusarium*. spp., such as the antibacterial fusapyridons, were not present among crude extracts of *F.* cf. *tricinctum*,^22^ but the diversity of bioactivity already documented in this class make these unknown compounds targets to pursue further. Although there were several additional nodes in the cluster contributed by *Ramularia* and *Plagiostoma* spp., none were dereplicated with the methods used.

### Cluster L—Griseofulvin

Within cluster L, griseofulvin and dechlorogriseofulvin were dereplicated by the GNPS library search function. The *M* + 2 node is present for griseofulvin and represents its ^37^Cl isotope, which lends further confidence to the identification. Two other nodes connected with griseofulvin have *m*/*z* values of 305.1019 [M + H]^+^ and 335.1125 [M + H]^+^, which correspond to formulas of C_16_H_16_O_6_ and C_17_H_18_O_7_, respectively. Griseophenone C is tentatively identified from the molecular formula C_16_H_16_O_6_, which shares a prominent product ion of *m*/*z* 165.0546 with griseofulvin and griseophenone B. The compound C_17_H_18_O_7_ shares this same product ion, but the formula does not match any previously published griseofulvin-related compounds. C_17_H_18_O_7_ is also connected to griseofulvin and dechlorogriseofulvin by high cosine scores of 0.94 and 0.93, respectively. Griseofulvin and dechlorogriseofulvin were present in most extracts of *X. ellisii* in the collection. Griseophenone C was present in five isolates of *X. ellisii*.

Griseofulvin is a known antifungal drug that is a common ingredient in topical antifungal creams and is used to treat a broad variety of human fungal infections. It also shows promise as a treatment for other conditions like gout and ischemic heart disease.^23^ Dechlorogriseofulvin also exhibits some antifungal activity, but to a lesser degree than its chlorinated analogue.^24,25^ Both compounds are known metabolites of *X. ellisii*.^19^ Griseophenone C has not been previously identified from *X. ellisii*, but it is a known precursor to griseofulvin within the biosynthetic pathway, so it is not unexpected to detect it along with other griseofulvin compounds. It has antibacterial activity against several bacterial species, including methicillin-resistant *S. aureus* and *E. coli*. No other griseophenones were identified in the network, although two unpaired nodes with *m*/*z* values matching those of griseophenone B (*m*/*z* 339.0629, C_16_H_15_ClO_6_) and griseophenone D (*m*/*z* 291.0863, C_15_H_14_O_6_) were present. The tandem mass spectrum collected at the *m*/*z* of griseophenone B also possessed the prominent product ion at *m*/*z* 165.0546 observed in the other griseofulvin-related compounds, and a ^37^Cl isotope pattern, suggesting a chlorine-containing griseofulvin relative. However, the tandem mass spectra for both features had fewer than ten fragment ions under these conditions and as such they did not form any connections with other nodes. Therefore, they were removed, and could not be conclusively dereplicated from the overall network.

### Cluster P—Hirsutatin A

**Figure 6:**
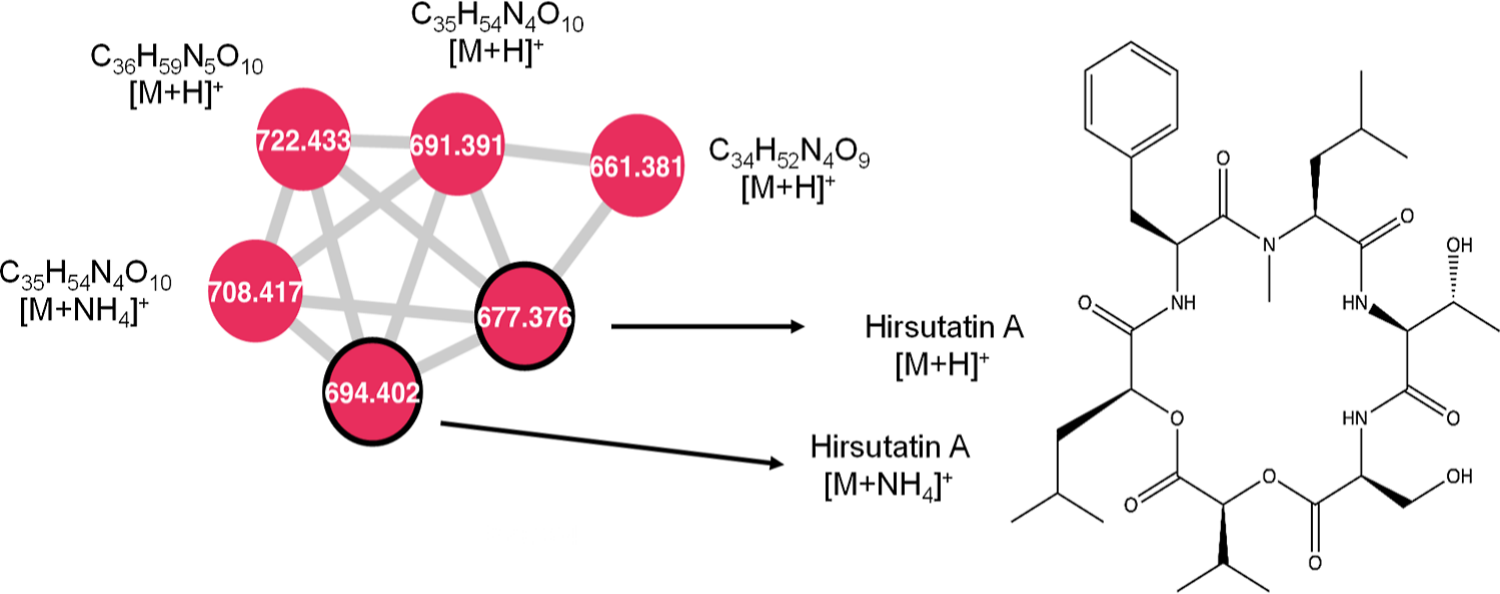
Close-up of cluster P from Figure 1. Hirsutatin A and related compounds are labeled above. All nodes were produced by isolates of *Xylaria*. Nodes outlined in black were included as seed spectra.

Hirsutatin A was dereplicated in extracts by comparing with the included seed spectrum of hirsutatin A. It was additionally present in one extract each of *Xylaria cubensis* and of *Godronia cassandrae*. Hirsutatin A is a cyclohexadepsipeptide originally identified from an insect pathogenic fungus and was later also observed as a natural product of the endophytic fungus *X. ellisii*. Within cluster P, it is present in both protonated and ammoniated forms. Hirsutatin A was connected with three unknown compounds with *m*/*z* values of 661.3804 [M + H]^+^, 691.3909 [M + H]^+^, and 722.4332 [M + H]^+^, corresponding to chemical formulas of C_34_H_52_N_4_O_9_, C_35_H_54_N_4_O_10_, and C_36_H_59_N_5_O_10_, respectively (Figure 6). These compounds are each connected to the hirsutatin A node with cosine similarity scores of 0.84, 0.88, and 0.95, respectively, indicating a high likelihood of structural similarity and did not match the formulas of any published depsipeptides.

Hirsutatin A has biological activity against *Mycobacterium tuberculosis*—the causative agent of tuberculosis in humans. Hirsutatin B was not present in the molecular network or the raw files, but it also has activity against *M. tuberculosis*, and strong activity against a multi-drug resistant strain of *Plasmodium falciparum*, which is the parasite responsible for malaria.^126^ Other cyclic depsipeptides also show a range of bioactivities, such as the enniatins produced by *Fusarium*, which are antibacterial, antihelminthic, insecticidal, antifungal, and herbicidal. Because of the bioactivities demonstrated by this compound class, the unidentified compounds noted in this cluster are worthwhile to investigate further.

### Unknown Clusters

As shown in Figure 1, many of the largest clusters did not contain any known compounds based on comparison to our seed spectra and the GNPS library. This does not necessarily indicate that they are a novel series of natural products, only that these spectra should be investigated in detail to determine if they represent tangible targets for purification and characterization. For example, by screening the strains represented in these clusters through bioactivity assays or by analyzing the existing extracts with negative mode ionization. In Figure 1, the eight largest unknown clusters (based on number of nodes) are listed numerically from U1–U8. Details about the detected analytes are listed in Table S3 and the three largest unknown clusters are discussed in more detail below.

#### Cluster U1

U1 is the largest cluster detected based on number of member nodes (Figure 1). A common spectral feature for many of the high intensity compounds of this cluster are the product ions of *m*/*z* 137.060 (C_8_H_9_O_2_^+^) and 109.0652 (C_7_H_9_O^+^). The major genus that produces compounds within this node is *Neocucurbitaria*.

**Figure 7:**
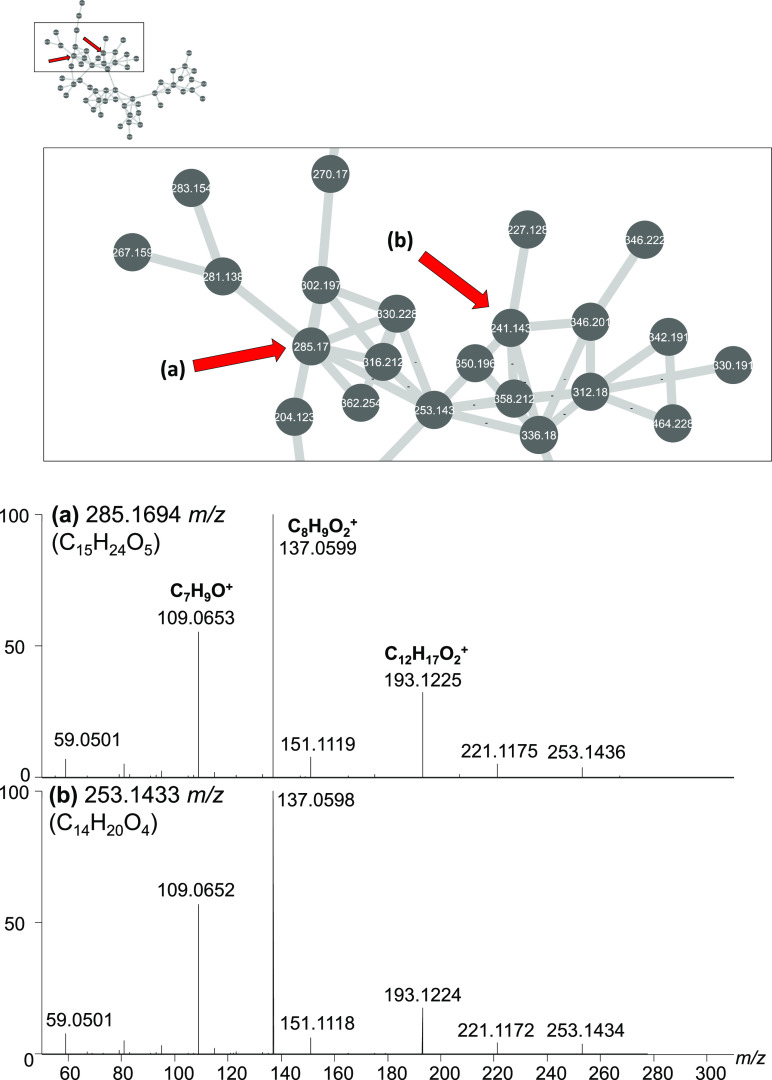
Close-up of cluster U1 from Figure 1. U1 is the largest cluster. Common molecular features include product ions at *m*/*z* C_7_H_9_O^+^, C_8_H_9_O_2_^+^, and C_12_H_17_O_2_^+^. The major producer of these compounds are *Neocucurbitaria* spp.

Two sample nodes from Cluster U1 are shown above in Figure 7. Chemical formulas were calculated from HRMS *m*/*z* values and were determined to be C_15_H_24_O_5_ and C_14_H_20_O_4_, neither of which yielded matches when compared to the GNPS library or seed spectra. Compounds within the cluster ranged in *m*/*z* from 170 to 527. There are no previously reported metabolites from *Neocucurbitaria* detectable within this cluster. Cluster U4 contains two nodes that match *m*/*z* values of neocucurbol A and neocucurbin G; however, there was no way to confirm the identities. Overall, there are few bioactive compounds reported from any *Neocucurbitaria*, making them interesting to investigate for new and novel compounds in the future.

#### Cluster U2

**Figure 8:**
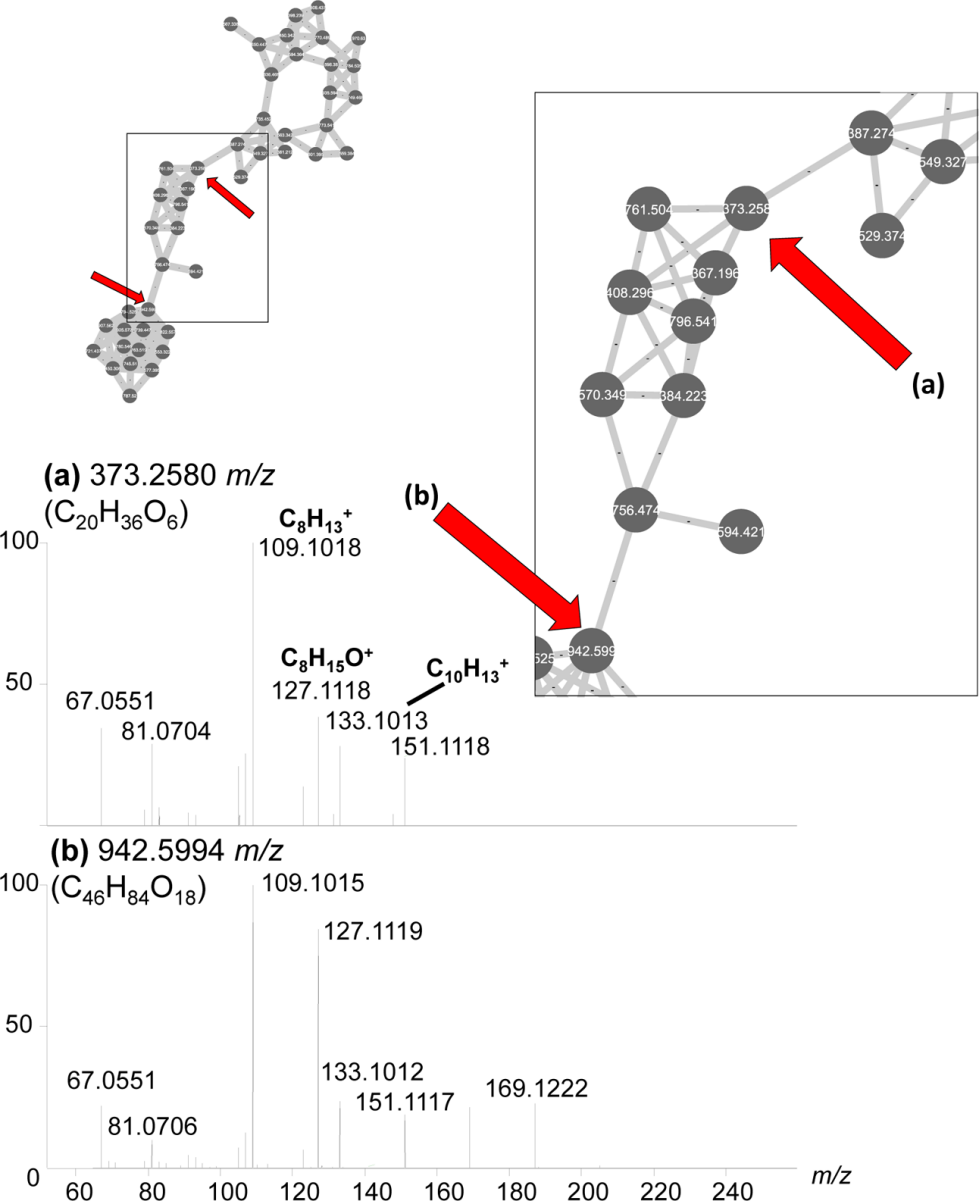
Close-up of cluster U2 from Figure 1. Molecular features within cluster U2 had common product ions at C_8_H_13_^+^, C_8_H_15_O^+^, and C_10_H_15_O^+^. At this collision energy, all major product ions are below *m*/*z* 200. The major producers of these compounds are *Seimatosporium* and *Nigrospora* spp.

Nodes in Cluster U2 are predominantly seen in the genera *Seimatosporium* and *Nigrospora*. Common product ions identified among several compounds in the cluster include *m*/*z* 109.1015 (C_8_H_13_^+^), 127.1119 (C_8_H_15_O^+^), and 133.1012 (C_10_H_13_^+^) (Figure 8).

Compounds in this cluster have *m*/*z* values ranging from 367 to 970 and all have formulas that contain only carbon, hydrogen, and oxygen. Both [M + H]^+^ and [M + NH_4_]^+^ adducts of compounds were present in many instances, as well as [M + H–H_2_O]^+^ ions. There were no other compounds present in the network previously reported from *Seimatosporium* or *Nigrospora* spp.; however, these are both understudied fungal species with few reported compounds to begin with, giving them a higher likelihood of producing new compounds.

#### Cluster U3

**Figure 9:**
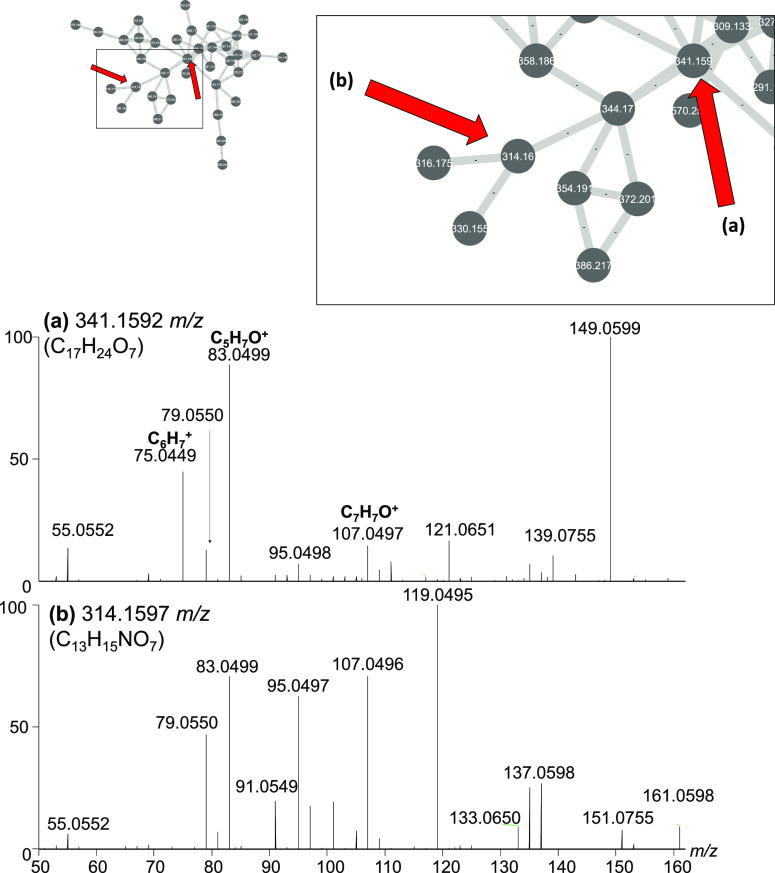
Close-up of cluster U3 from Figure 1. Molecular features within cluster U3 had common product ions at C_6_H_7_^+^, C_5_H_7_O^+^, and C_7_H_7_O^+^. The major producers of these compounds are *Diaporthe* spp. and a species from the order *Xylariales*.

The major contributor to nodes within cluster U3 are species from the genus *Diaporthe*, as well as an undefined species from the order Xylariales (Figure 9).

Compounds in this cluster ranged from *m*/*z* 281 to 570 and are composed of carbon, hydrogen, and oxygen, while some also contain nitrogen. There were no other identifiable compounds previously reported from *Diaporthe* spp. in the network.

### Seed Spectra

Of the previously isolated compounds that were included as seed spectra, several were not identifiable within the final network, namely coriloxin, abscisic acid, aschochitine, 4,10-dihydro-3,7,8-trihydroxy-3-methyl-10-oxo-1*H*,3*H*-pyrano[4,3-*b*][1]benzopyran-9-carboxylic acid, and 7-hydroxy-3-(hydroxymethyl)-2-(2-hydroxypropyl)-6-methoxy-4*H*-chromen-4-one. These were present in the mass spectra of fungi that are known to produce them, however upon closer inspection, either they had too few fragments to form connections to other nodes or there were no additional compounds similar enough to connect with.

Other seed spectra were present within the network but did not connect to any novel compounds. Cytochalasin D was dereplicated within *X. ellisii* extracts and was present within cluster K but was not connected with any new compounds. Epoxycytochalasin D and zygosporin E were both dereplicated within cluster B from extracts of *X. ellisii*, along with several other cytochalasins, however, no new compounds were present (Table 1).

**Table 1:** Unknown Compounds Identified from Canadian Fungal Endophytes as Targets for Isolation and Characterization

formula	[M + H]^+^ *m*/*z*	RT (min)	mass error (Δppm)	structurally related to	produced by
C_17_H_18_O_7_	335.1125	3.41	–0.117	griseofulvin	*X. ellisii*
C_28_H_39_NO_3_	438.3001	5.79	–0.298	oxysporidinone	*Fusarium* cf. *tricinctum*
C_29_H_45_NO_6_	504.3321	5.07	0.189	oxysporidinone	*Fusarium* cf. *tricinctum*
C_29_H_51_N_5_O_6_	566.3914	4.08	0.369	ellisiiamides	*X. ellisii*
C_34_H_52_N_4_O_9_	661.3804	4.60	–0.507	hirsutatin A	*X. ellisii*
C_35_H_54_N_4_O_10_	691.3909	4.48	–0.044	hirsutatin A	*X. ellisii*
C_36_H_59_N_5_O_10_	722.4332	4.37	–0.414	hirsutatin A	*X. ellisii*

## Conclusions

Ultimately, the use of molecular networking as a dereplication and processing strategy with a large dataset of 288 fungal endophyte extracts was successful in identifying seven unknown compounds as targets for isolation that are strong candidates for bioactivity due to their relatedness to known potent bioactive compounds. These include relatives from diverse chemical classes, namely, one griseofulvin relative, three hirsutatin-related compounds, a new ellisiiamide, and two new sambutoxin- and oxysporidinone-related compounds.

A challenge of molecular networking as a dataset mining strategy is that it is more successful when samples contain multiple compounds with similar chemical structures. If a dataset contains few structurally related compounds, this approach will overlook potentially valuable compounds that have no neighbors within the molecular network. Luckily, most biosynthetic pathways generate mixtures of structurally related compounds. While molecular networking may not be able to assign relationships to all compounds within a sample, it is becoming a more powerful dereplication tool, especially as databases improve, thus rapidly speeding up the analysis of large tandem mass spectra datasets.

Future works will focus on purifying the targeted new compounds for structural characterization and biological activity assessments. The fungal endophytes will ideally be employed as part of an integrated crop management strategy in their host plants. The use of endophyte-enhanced plants in agriculture will help meet the Government of Canada’s mandate to reduce our reliance on chemical pesticides.

## Materials and Methods

### Endophyte Sampling and Extraction

Leaves and stems from Canadian fruit bearing crops, including blueberries, cranberries, raspberries, and grapes, were sampled in Ontario, New Brunswick, and Nova Scotia, Canada, between 2011 and 2015 (Supporting Information Table S1). Endophytes were isolated from these plants following the method provided by Ginn (1998).^127^ In short, leaves and stems were surface-sterilized with bleach and ethanol, then were sliced into 1 cm pieces and placed on potato dextrose agar (PDA) media (Sigma-Aldrich, MO, USA). Fungal samples that grew from plant material were extracted with an UltraClean Microbial DNA Isolation Kit (MoBio, Carlsbad, CA). Polymerase chain reactions (PCR) were performed in an Eppendorf Mastercycler Nexus Gradient Thermal Cycler. The total volume of the reaction was 25 μL, consisting of 22.5 μL of Platinum Blue SuperMix (Invitrogen, Carlsbad, CA, USA), 1 μL of genomic DNA (15 ng/μL), and 0.75 μL each of forward and reverse primers (ITS1 and ITS4, respectively) for a final concentration of 0.3 μM. PCR conditions include an initial denaturation at 94 °C for 2 min, then 35 cycles of 1 min at 94 °C, 30 s at 60 °C, and 30 s at 72 °C, then a final elongation of 5 min at 72 °C. DNA sequences were interpreted using NCBI BLAST blastn suite (https://blast.ncbi.nlm.nih.gov/). Isolated endophytes were grown on 20 mL of PDA at 23 °C for 2–6 weeks or until cells reached confluence. Cultures were extracted by homogenizing the agar and cells with 20 mL of methanol (Fisher Scientific, NJ, USA) and then gravity filtering the homogenate with a no. 1 Whatman filter. One-milliliter aliquots of filtrate were taken, then dried at room temperature under nitrogen. Dried samples were stored at −20 °C until analysis.

### Seed Spectra

Seed spectra included in analysis are presented in Table 2. These were previously isolated by HPLC and characterized by NMR as described in Ibrahim (2017). Seed spectra compounds were analyzed by LC–MS/MS alongside endophyte extracts.

**Table 2:** Seed Spectra Used in Molecular Network of Canadian Fungal Endophytes^a^

name	calc *m*/*z* [M + H]^+^	formula	fungal source
coriloxin	171.0652	C_8_H_10_O_4_	*X. castorea*
abscisic acid	265.1434	C_15_H_20_O_4_	*N. sphaerica*
ascochitine	277.1071	C_15_H_16_O_5_	*Coniochaeta* cf. *marina*
7-hydroxy-3-(hydroxymethyl)-2-(2-hydroxypropyl)-6-methoxy-4*H*-chromen-4-one (fulvic acid derivative)	281.1019	C_14_H_16_O_6_	*S. vaccinii*
4,10-Dihydro-3,7,8-trihydroxy-3-methyl-10-oxo-1*H*,3*H*-pyrano[4,3-*b*][1]benzopyran-9-carboxylic acid (fulvic acid analogue)	309.0605	C_14_H_12_O_8_	*S. vaccinii*
zygosporin E	492.2744	C_30_H_37_NO_5_	*X. ellisii*
cytochalasin D	508.2693	C_30_H_37_NO_6_	*X. ellisii*
epoxycytochalasin D	524.2642	C_30_H_37_NO_7_	*X. ellisii*
ellisiiamide A	556.3493	C_30_H_45_N_5_O_5_	*X. ellisii*
ellisiiamide B	570.3650	C_31_H_47_N_5_O_5_	*X. ellisii*
cyclic pentapeptide 1	584.3806	C_32_H_49_N_5_O_5_	*X. ellisii*
ellisiiamide C	598.3963	C_33_H_51_N_5_O_5_	*X. ellisii*
ellisiiamide G	600.3755	C_32_H_49_N_5_O_6_	*X. ellisii*
hirsutatin A	677.3756	C_34_H_52_N_4_O_10_	*X. ellisii*

a Seed spectra included in the network are bioactive compounds previously isolated from fungal endophytes.

### Analysis by LC–HRMS/MS

In preparation for analysis, samples and seed spectra were reconstituted in 1 mL of methanol (Fisher Scientific, Fair Lawn, NJ, USA) and analyzed by LC–HESI–HRMS/MS on a Thermo Q-Exactive Orbitrap mass spectrometer paired with an Agilent 1290 UHPLC system. Additionally, pooled QC samples were prepared by combining 10 μL aliquots from each sample.

Chromatographic separation was accomplished using a dual-solvent system with acetonitrile + 0.1% formic acid (solvent A, Fisher Scientific, Fair Lawn, NJ, USA) and water + 0.1% formic acid (solvent B, Fisher Scientific, Fair Lawn, NJ, USA) at a rate of 0.3 mL/min. The gradient was held at 0% B for 0.5 min, increased to 100% B over 3 min, held at 100% B for 2.5 min, then decreased to 0% B over 0.5 min and held at 0% B for 1 min. All samples were injected in 5 μL portions on an EclipsePlus RRHD C-18 column (2.1 × 50 mm, 1.8 μm; Agilent) that was maintained at 35 °C. Heated electrospray ionization (HESI) conditions were as follows: capillary temperature, 400 °C; sheath gas, 17 units; auxiliary gas, 8 units; probe heater temperature, 450 °C; S-Lens RF level, 50; and capillary voltage, 3.9 kV.

Data were acquired in positive ionization mode with data-dependent acquisition with the following settings: resolution, 70,000; automatic gain control (AGC) target, 1 × 10^6^; max IT, 256 ms; scan range, 100–1500 *m*/*z*. The 10 ions with highest intensity from each MS scan were selected to be fragmented by MS/MS at resolution 17,500; AGC target 1 × 10^6^; max IT, 64 ms; stepped NCE, 28/50; isolation window, 1.2 *m*/*z*; intensity threshold, 1.3 × 10^5^; dynamic exclusion, 10.0 s. QC samples were injected at the beginning, end, and throughout the run to assess instrument drift.

### Data Processing and Principal Component Analysis

Thermo Raw files were converted to mzML format using MSConvert (v. 3) with the following settings: 32 bit binary encoding precision, no file compression, and peak picking from levels 1 to 2.^128^ Converted files were brought into R (3.5.3) to perform principal component analysis (PCA) with the packages xcms (3.2.0), FactoMineR (2.3), and MetabolAnalyze (1.3.1).^128−27^ Settings for processing in R were as follows: method, centWave; prefilter, (5, 5000); ppm, 5; snthresh, 10; peakwidth, (5, 20); noise, 500,000; bw, 5; minfrac, 0.001; and mzwid, 0.015. Zero values were imputed with two-thirds of the lowest value measured for each metabolite. Peak area values were log-10 transformed and pareto scaled. PCA was performed with only QC samples to assess instrument drift over the course of analysis. The first and second principal components were plotted against one another.

### GNPS Parameters and Processing

Converted mzML files were also uploaded to the GNPS molecular networking site with FileZilla (3.9.0.5) and analyzed with the following settings: precursor ion mass tolerance, 0.02 Da; fragment ion mass tolerance, 0.02 Da; min pairs cosine, 0.75; network topK, 10; maximum connected component size, 100; minimum matched fragment ions, 5; minimum cluster size, 2; and MSCluster, on. Network output was downloaded as a GRAPHML file and was imported into Cytoscape (3.6.1) for visualization. To simplify network analysis, all unconnected nodes were removed, along with nodes attributed to blank media, clusters formed solely from seed spectra, and background ions.

Files were also assessed with the Library Search function of GNPS to dereplicate compounds. The following settings were used: precursor ion mass tolerance, 0.02 Da; fragment ion mass tolerance, 0.02 Da; min matched fragment ions, 5; and cut-off score, 0.75. Additional compounds were dereplicated by comparing to an in-house database of MS/MS spectra.

## Supporting Information Available

PCA plot performed to assess instrument performance, additional views of unknown clusters from the molecular network, all endophyte isolates examined in this study, and identities of all dereplicated compounds present in the network (PDF)
Precursor mass, retention times, calculated formulas, unique file sources, and putative identifications of all features from their corresponding clusters (XLSX)

## Notes

Data available at the Metabolomics Repository with Study ID number ST002482.
