# Supplementary material for: Non-targeted Screening of Natural Products from 288 Fungal Endophytes from Canadian Fruit Crops
Source: ACS Omega. 2023 Jun 27;8(27):24561–72. doi: 10.1021/acsomega.3c02786 (PMC10339433; doi:10.1021/acsomega.3c02786)
Supplement: Supplementary file 1 — ao3c02786_si_001.pdf [file ao3c02786_si_001.pdf]

Non-targeted screening of natural products from 288 fungal endophytes from Canadian fruit crops

Natasha DesRochers[1,2], Justin B. Renaud[1], Joey B. Tanney[3], Ashraf Ibrahim[4], Ken K.-C. Yeung[2,5], Mark W. Sumarah[1,2]*

[1] London Research and Development Centre, Agriculture and Agri-Food Canada, 1391 Sandford St., London, Ontario, Canada, N5V4T3
[2] Department of Chemistry, University of Western Ontario, 1151 Richmond St., London, Ontario, Canada, N6A 3K7
[3] Natural Resources Canada, Pacific Forestry Centre, 506 Burnside Road, Victoria, British Columbia, Canada, V8Z 1M5
[4] McMaster University, 1290 Main St. W, Hamilton, Ontario, Canada, L8S 4L8
[5] Department of Biochemistry, University of Western Ontario, 1151 Richmond St., London, Ontario, Canada, N6A 3K7

***** Correspondence: mark.sumarah@agr.gc.ca; Tel.: +01-226-688-6187

Supplementary Materials:

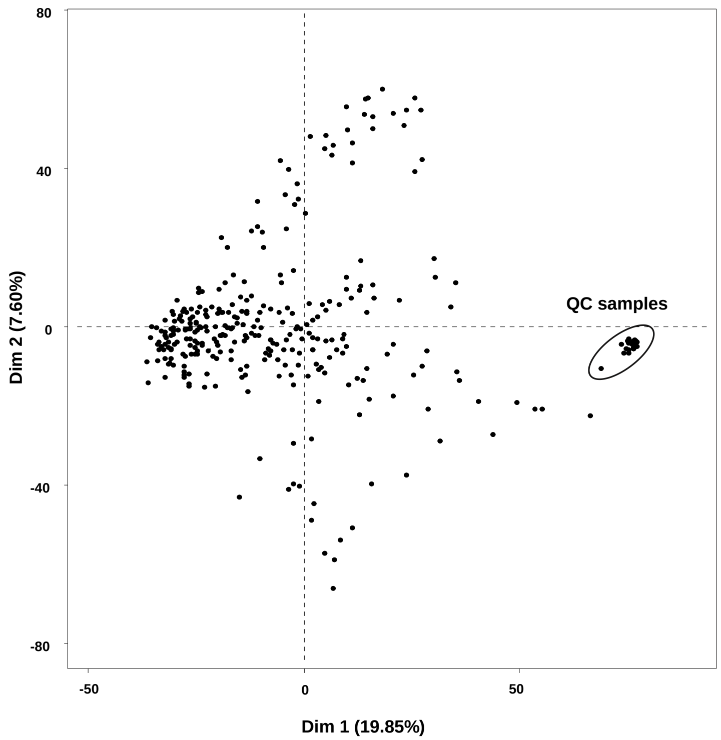

Figure S1. PCA plot of endophyte extracts and QC samples in positive ionization mode.

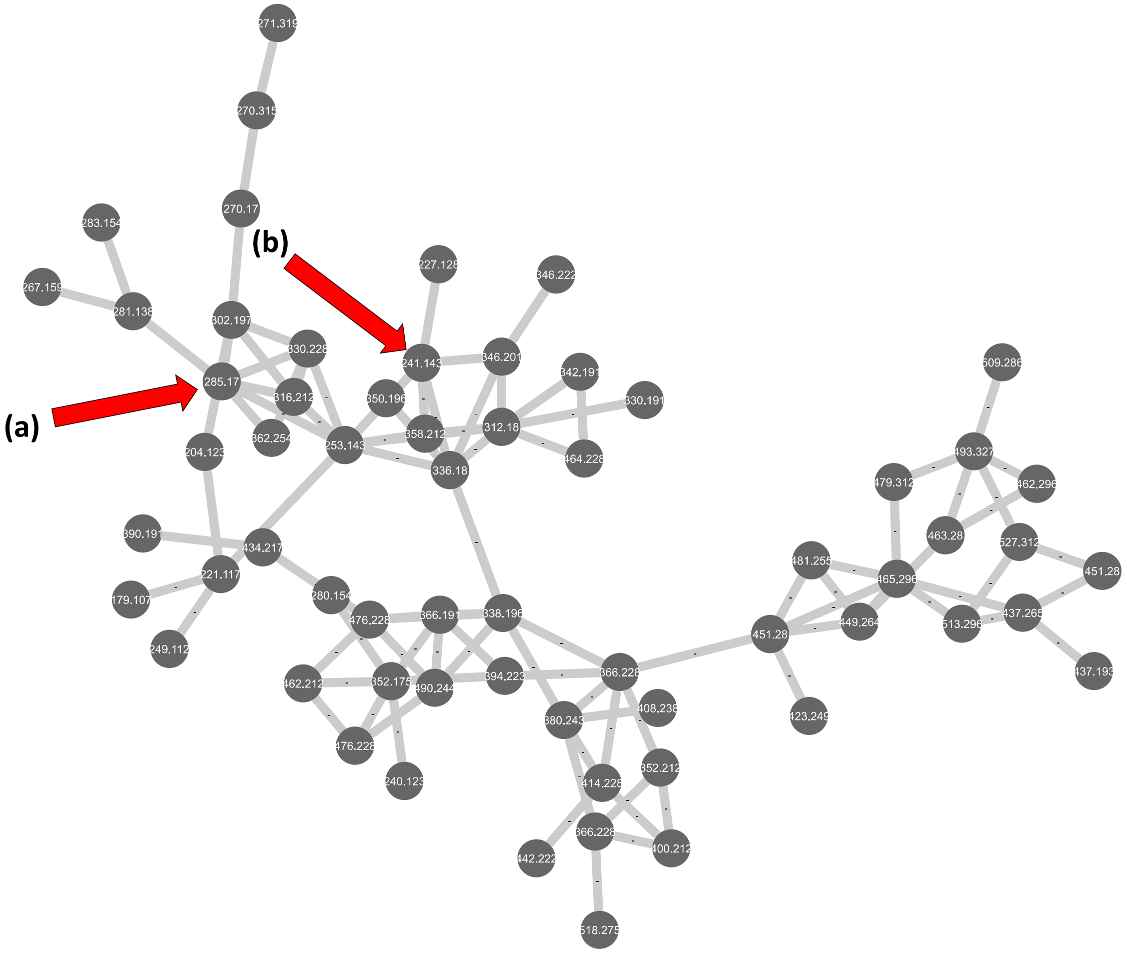

Figure S2. Enlarged version of unknown cluster U1 pictured in Figure 1.

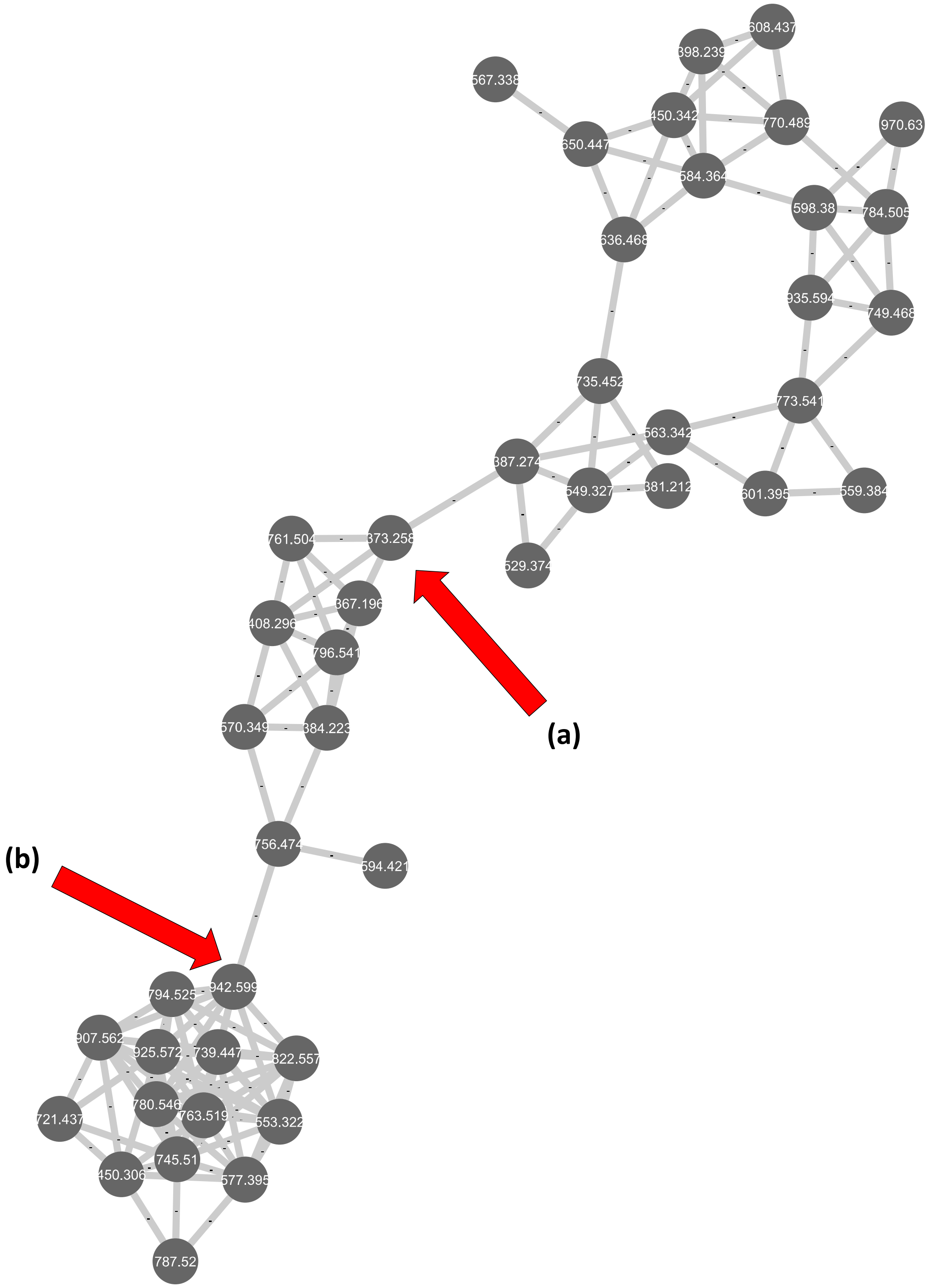

Figure S3. Enlarged version of unknown cluster U2 pictured in Figure 1.

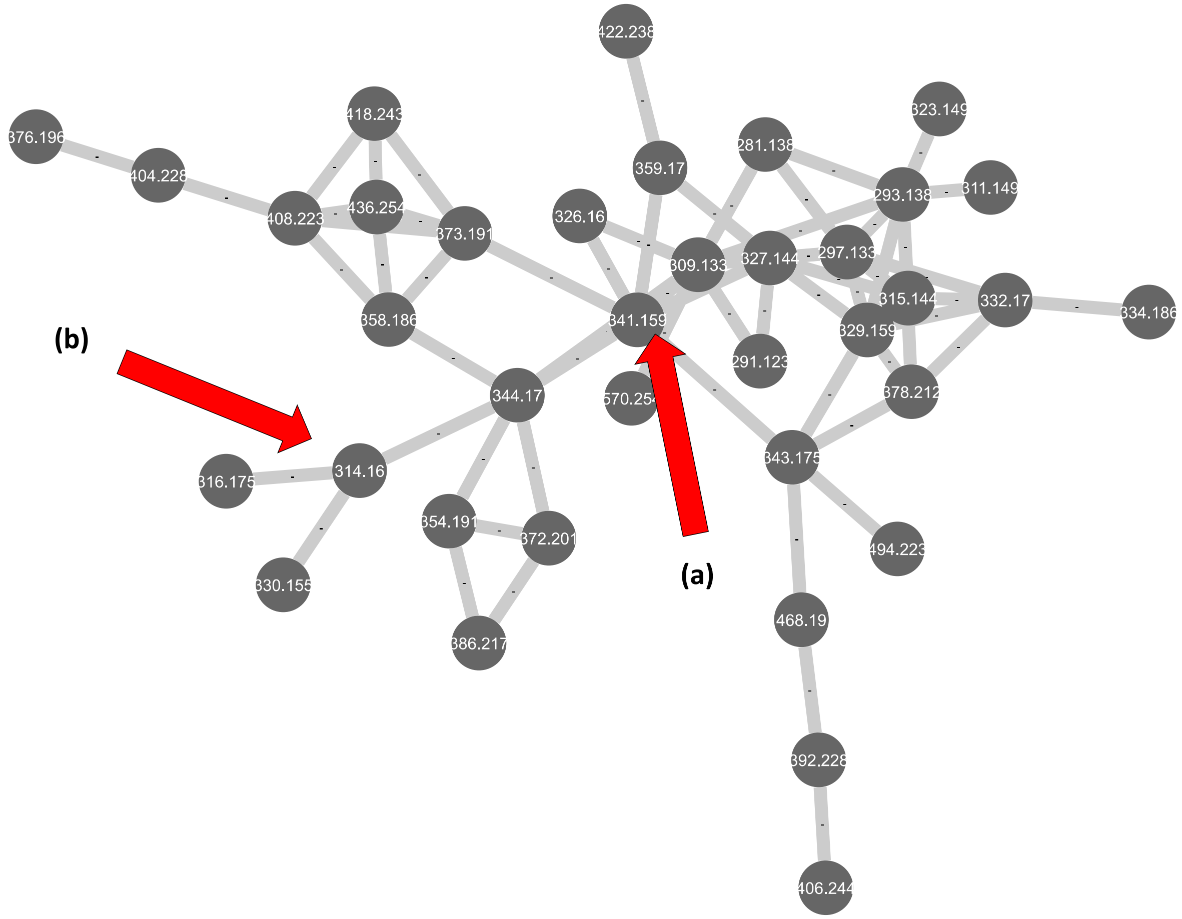

Figure S4. Enlarged version of unknown cluster U3 pictured in Figure 1.

Table S1. List of endophyte isolates used for GNPS analysis, with tentative species identification based on ITS sequencing, geographic origin, and host organism listed where available.

Endophyte ID	Tentative species ID	Origin	Host Organism
E-002	*Epicoccum nigrum*	Bala, ON	Cranberry
E-003	*Setomelanomma holmii*	Bala, ON	Cranberry
E-004	*Botrytis cinerea*	Bala, ON	Cranberry
E-005	*Epicoccum nigrum*	Bala, ON	Cranberry
E-006	*Nigrospora sphaerica*	Bala, ON	Cranberry
E-007	*Articulospora atra*	Bala, ON	Cranberry
E-008	*Nemania serpens*	Bala, ON	Cranberry
E-009	*Venturia* sp.	Bala, ON	Cranberry
E-010	*Venturia* sp.	Bala, ON	Cranberry
E-011	*Seimatosporium lichenicola*	Debert, NS	Lowbush blueberry
E-012	Dothideaceae sp.	Debert, NS	Lowbush blueberry
E-015	*Paramycosphaerella* sp.	Portapique, NS	Lowbush blueberry
E-016	*Didymella* sp.	Portapique, NS	Lowbush blueberry
E-017	*Alpinaria* cf. *rhododendri*	Portapique, NS	Lowbush blueberry
E-018	*Chaetopsis* sp.	Portapique, NS	Lowbush blueberry
E-019	*Alpinaria* cf. *rhododendri*	Portapique, NS	Lowbush blueberry
E-020	*Phyllosticta vaccinii*	Portapique, NS	Lowbush blueberry
E-021	Dothideaceae sp.	Portapique, NS	Lowbush blueberry
E-022	Dothideaceae sp.	Mt. Thom, NS	Lowbush blueberry
E-023	Dothideaceae sp.	Mt. Thom, NS	Lowbush blueberry
E-024	*Leptodontidium* sp.	Mt. Thom, NS	Lowbush blueberry
E-025	Dothideaceae sp.	Mt. Thom, NS	Lowbush blueberry
E-026	*Coniochaeta* cf. *marina*	Mt. Thom, NS	Lowbush blueberry
E-027	Dothideaceae sp.	Mt. Thom, NS	Lowbush blueberry
E-028	*Xylaria ellisii*	Mt. Thom, NS	Lowbush blueberry
E-029	Dothideaceae sp.	Mt. Thom, NS	Lowbush blueberry
E-030	*Phialocephala* sp.	Mt. Thom, NS	Lowbush blueberry
E-031	*Phialocephala* sp.	Mt. Thom, NS	Lowbush blueberry
E-032	*Godronia* cf. *cassandrae*	Mt. Thom, NS	Lowbush blueberry
E-033	Dothideaceae sp.	Bala, ON	Cranberry
E-036	*Godronia* cf. *cassandrae*	Mt. Thom, NS	Lowbush blueberry

Endophyte ID	Tentative species ID	Origin	Host Organism
E-038	Dothideaceae sp.	Bala, ON	Cranberry
E-039	*Mollisia* cf. *melaleuca*	Mt. Thom, NS	Lowbush blueberry
E-040	*Mollisia* cf. *nigrescens*	Simcoe, ON	Highbush blueberry
E-041	*Casaresia* cf. *sphagnorum*	Mt. Thom, NS	Lowbush blueberry
E-042	*Pyrenopeziza* sp.	Portapique, NS	Lowbush blueberry
E-043	*Lachnum* sp.	Simcoe, ON	Highbush blueberry
E-044	*Phialocephala* sp.	Mt. Thom, NS	Lowbush blueberry
E-045	*Godronia cassandrae*	Mt. Thom, NS	Lowbush blueberry
E-046	*Godronia cassandrae*	Portapique, NS	Lowbush blueberry
E-047	*Paramycosphaerella* sp.	Portapique, ON	Lowbush blueberry
E-048	*Discosia rubi*	Parks Blueberries, ON	Highbush blueberry
E-049	*Phyllosticta pyrolae*	Mt. Thom, NS	Lowbush blueberry
E-050	*Nemania serpens*	Simcoe, ON	Highbush blueberry
E-051	*Coniochaeta* cf. *marina*	Portapique, ON	Lowbush blueberry
E-052	*Paramycosphaerella* sp.	Portapique, ON	Lowbush blueberry
E-053	*Leptodontidium* sp.	Mt. Thom, NS	Lowbush blueberry
E-054	*Phyllosticta vaccinii*	P.E.I.	Lowbush blueberry
E-055	Dothideaceae sp.	Bala, ON	Cranberry
E-056	Dothideaceae sp.	Bala, ON	Cranberry
E-058	*Kretzschmaria hedjaroudei*	Jordan Station, ON	Grape
E-059	*Setomelanomma holmii*	Bala, ON	Cranberry
E-060	Dothideaceae sp.	Mt. Thom, NS	Lowbush blueberry
E-062	*Diaporthe phaseolorum*	Simcoe, ON	Highbush blueberry
E-063	*Mollisia* cf. *melaleuca*	Portapique, NS	Lowbush blueberry
E-064	Barbatosphaeriaceae sp.	Jordan Station, ON	Highbush blueberry
E-065	*Seimatosporium lichenicola*	Jordan Station, ON	Highbush blueberry
E-066	*Didymella pomorum*	Parks Blueberries, ON	Highbush blueberry
E-067	*Geastrumia polystigmatis*	Mt. Thom, NS	Lowbush blueberry
E-068	*Articulospora atra*	Bala, ON	Cranberry
E-069	Dothideaceae sp.	Debert, NS	Lowbush blueberry
E-070	Dothideaceae sp.	Debert, NS	Lowbush blueberry
E-072	*Mollisia* cf. *melaleuca*	Bala, ON	Cranberry

**Endophyte ID**	**Tentative species ID**	**Origin**	**Host Organism**
E-073	*Sphaerulina vaccinii*	Debert, NS	Lowbush blueberry
E-075	*Xylaria corniformis*	Jordan Station, ON	Grape
E-076	*Lachnum* sp.	Bala, ON	Cranberry
E-077	*Lophiotrema mucilaginosis*	Debert, NS	Lowbush blueberry
E-079	*Neovaginatispora* sp.	Mt. Thom, NS	Lowbush blueberry
E-081	Dothideaceae sp.	Debert, NS	Lowbush blueberry
E-082	*Hypomontagnella submonticulosa*	Jordan Station, ON	Raspberry
E-083	Rhytismataceae sp.	Portapique, NS	Lowbush blueberry
E-084	*Hypomontagnella submonticulosa*	Jordan Station, ON	Pear
E-086	*Godronia cassandrae*	Portapique, NS	Lowbush blueberry
E-087	*Coniochaeta* cf. *marina*	Portapique, NS	Lowbush blueberry
E-088	*Phyllosticta pyrolae*	Mt. Thom, NS	Lowbush blueberry
E-089	*Godronia cassandrae*	Portapique, NS	Lowbush blueberry
E-090	Hyaloscyphaceae sp.	Portapique, NS	Lowbush blueberry
E-091	*Sphaerulina vaccinii*	Portapique, NS	Lowbush blueberry
E-092	*Trichaptum abietinum*	Debert, NS	Lowbush blueberry
E-093	*Phaeomoniellales* sp.	Bala, ON	Cranberry
E-094	*Godronia cassandrae*	Debert, NS	Lowbush blueberry
E-095	*Leptodontidium* sp.	Mt. Thom, NS	Lowbush blueberry
E-096	*Leptodontidium* sp.	Portapique, NS	Lowbush blueberry
E-097	*Microdochium neoqueenslandicum*	Rawdon, NS	Lowbush blueberry
E-099	*Pseudoplectania nigrella*	Jordan Station, ON	Grape
E-100	*Mollisia* cf. *nigrescens*	Mt, Thom, NS	Lowbush blueberry
E-101	*Creosphaeria sassafras*	Jordan Station, ON	Grape
E-102	*Leptodontidium* sp.	Portapique, NS	Lowbush blueberry
E-103	*Nemania serpens*	Rawdon, NS	Highbush blueberry
E-104	*Xylaria ellisii*	Rawdon, NS	Highbush blueberry
E-105	*Godronia cassandrae*	Mt. Thom, NS	Lowbush blueberry
E-106	Tympanidaceae sp.	Mt. Thom, NS	Lowbush blueberry
E-107	*Xylaria ellisii*	Mt. Thom, NS	Lowbush blueberry
E-108	*Leptodontidium* sp.	Mt. Thom, NS	Lowbush blueberry

Endophyte ID	Tentative species ID	Origin	Host Organism
E-109	*Mollisia* cf. *melaleuca*	Mt. Thom, NS	Lowbush blueberry
E-110	Tympanidaceae sp.	Mt. Thom, NS	Lowbush blueberry
E-111	*Hypoderma* cf. *rubi*	Debert, NS	Lowbush blueberry
E-112	*Xylaria ellisii*	Mt. Thom, NS	Lowbush blueberry
E-113	*Leptodontidium* sp.	Mt. Thom, NS	Lowbush blueberry
E-114	*Xylaria ellisii*	Debert, NS	Lowbush blueberry
E-115	*Xylaria ellisii*	Rawdon, NS	Highbush blueberry
E-116	*Xylaria ellisii*	Portapique, NS	Lowbush blueberry
E-117	*Pilidium concavum*	Portapique, NS	Lowbush blueberry
E-118	*Pilidium concavum*	Portapique, NS	Lowbush blueberry
E-119	*Mollisia* cf. *melaleuca*	Debert, NS	Lowbush blueberry
E-120	Dothideaceae sp.	Portapique, NS	Lowbush blueberry
E-121	PCR unsuccessful	Portapique, NS	Lowbush blueberry
E-122	*Alternaria alternata*	Mt. Thom, NS	Lowbush blueberry
E-123	*Xylaria ellisii*	Rawdon, NS	Highbush blueberry
E-124	*Mollisia* cf. *nigrescens*	Portapique, NS	Lowbush blueberry
E-125	*Alternaria arborescens*	Jordan Station, ON	Raspberry
E-129	*Xylaria ellisii*	Portapique, NS	Lowbush blueberry
E-130	*Hypoderma* cf. *rubi*	Debert, NS	Lowbush blueberry
E-133	Neodevriesiaceae sp.	Mt. Thom, NS	Lowbush blueberry
E-135	*Sphaerulina vaccinii*	Mt. Thom, NS	Lowbush blueberry
E-138	*Xylaria ellisii*	Rawdon, NS	Highbush blueberry
E-140	*Nemania serpens*	Jordan Station, ON	Grape
E-141	Dothideaceae sp.	Mt. Thom, NS	Lowbush blueberry
E-142	*Xylaria ellisii*	Portapique, NS	Lowbush blueberry
E-143	*Xylaria ellisii*	Portapique, NS	Lowbush blueberry
E-144	*Alpinaria* cf. *rhododendri*	Rawdon, NS	Highbush blueberry
E-145	*Alternaria arborescens*	Jordan Station, ON	Raspberry
E-148	*Nemania* sp.	Jordan Station, ON	Grape
E-150	*Xylaria ellisii*	Mt. Thom, NS	Lowbush blueberry
E-152	Dothideaceae sp.	Portapique, NS	Lowbush blueberry
E-153	*Xylaria ellisii*	Portapique, NS	Lowbush blueberry
E-154	*Mollisia* cf. *nigrescens*	Portapique, NS	Lowbush blueberry
E-155	*Leptodontidium* sp.	Portapique, NS	Lowbush blueberry

**Endophyte ID**	**Tentative species ID**	**Origin**	**Host Organism**
E-156	*Nemania diffusa*	Jordan Station, ON	Highbush blueberry
E-157	*Xylaria ellisii*	Portapique, NS	Lowbush blueberry
E-158	Dothideaceae sp.	Portapique, NS	Lowbush blueberry
E-159	*Nemania* sp.	Portapique, NS	Lowbush blueberry
E-160	*Seimatosporium lichenicola*	Portapique, NS	Lowbush blueberry
E-161	Dothideaceae sp.	Portapique, NS	Lowbush blueberry
E-162	*Xylaria ellisii*	Rawdon, NS	Highbush blueberry
E-163	*Aureobasidium* cf. *subglaciale*	Rawdon, NS	Highbush blueberry
E-164	*Xylaria ellisii*	Debert, NS	Lowbush blueberry
E-165	*Phaeoacremonium fraxinopennsylvanicum*	Rawdon, NS	Highbush blueberry
E-167	*Xylaria ellisii*	Debert, NS	Lowbush blueberry
E-168	*Mollisia* cf. *melaleuca*	Debert, NS	Lowbush blueberry
E-169	*Alternaria arborescens*	Jordan Station, ON	Raspberry
E-170	*Xylaria ellisii*	Mt. Thom, NS	Lowbush blueberry
E-171	*Alternaria infectoria*	Jordan Station, ON	Highbush blueberry
E-172	*Anthostomella* sp.	Jordan Station, ON	Grape
E-173	*Nemania serpens*	Jordan Station, ON	Raspberry
E-174	*Diaporthe vaccinii*	Mt. Thom, NS	Blueberry
E-175	*Phyllosticta* cf. *pyrolae*	Debert, NS	Blueberry
E-177	*Paraphaeosphaeria neglecta*	Mt. Thom, NS	Blueberry
E-178	*Fusarium tricinctum*	Mt. Thom, NS	Blueberry
E-179	*Sphaerulina vaccinii*	Debert, NS	Blueberry
E-180	*Diaporthe vaccinii*	Debert, NS	Blueberry
E-181	*Epicoccum nigrum*	Debert, NS	Blueberry
E-182	*Paraphaeosphaeria neglecta*	Mt. Thom, NS	Blueberry
E-183	*Godronia cassandrae*	Portapique, NS	Blueberry
E-184	*Xylaria cubensis*	Simcoe, ON	Grape
E-188	Dothideaceae sp.	Mt. Thom, NS	Blueberry
E-189	*Paraphaeosphaeria neglecta*	Mt. Thom, NS	Blueberry
E-190	*Sphaerulina vaccinii*	Mt. Thom, NS	Blueberry
E-191	Dothideaceae sp.	Mt. Thom, NS	Blueberry
E-192	*Sphaerulina vaccinii*	Mt. Thom, NS	Blueberry
E-193	Dothideaceae sp.	Mt. Thom, NS	Blueberry

Endophyte ID	Tentative species ID	Origin	Host Organism
E-194	*Nemania serpens*	Jordan Station, ON	Grape
E-196	*Mollisia* cf. *nigrescens*	Portapique, NS	Blueberry
E-197	*Cryptosporella* sp.	Portapique, NS	Blueberry
E-198	*Diaporthe vaccinii*	Mt. Thom, NS	Blueberry
E-199	*Kretzschmaria hedjaroudei*	Jordan Station, ON	Blueberry
E-200	*Fusarium tricinctum*	Mt. Thom, NS	Blueberry
E-201	Tympanidaceae sp.	Debert, NS	Blueberry
E-202	*Nemania serpens*	Jordan Station, ON	Grape
E-203	*Coniothyrium ferrarisianum*	Jordan Station, ON	Grape
E-204	*Phyllosticta* cf. *pyrolae*	Mt. Thom, NS	Blueberry
E-205	*Godronia cassandrae*	Mt. Thom, NS	Blueberry
E-206	*Xylaria ellisii*	Portapique, NS	Blueberry
E-207	*Seimatosporium lichenicola*	New Brunswick, CA	Blueberry
E-208	*Xylaria ellisii*	New Brunswick, CA	Blueberry
E-209	*Godronia cassandrae*	New Brunswick, CA	Blueberry
E-210	*Godronia cassandrae*	New Brunswick, CA	Blueberry
E-211	*Godronia cassandrae*	New Brunswick, CA	Blueberry
E-212	*Hypoderma* cf. *rubi*	New Brunswick, CA	Blueberry
E-213	*Godronia cassandrae*	New Brunswick, CA	Blueberry
E-214	*Hypoderma* cf. *rubi*	New Brunswick, CA	Blueberry
E-215	*Ramularia* sp.	New Brunswick, CA	Blueberry
E-216	*Xylaria ellisii*	New Brunswick, CA	Blueberry
E-217	*Mollisia* cf. *nigrescens*	Portapique, NS	Blueberry
E-218	*Mollisia* sp.	New Brunswick, CA	Blueberry
E-219	*Stemphylium globuliferum*	Debert, NS	Blueberry
E-220	*Camaropella* sp.	Jordan Station, ON	Grape
E-221	*Leptodontidium* sp.	Portapique, NS	Blueberry
E-222	*Pleosporales* sp.	Mt. Thom, NS	Blueberry
E-223	*Nemania serpens*	Debert, NS	Blueberry
E-224	*Paraphaeosphaeria neglecta*	Mt. Thom, NS	Blueberry
E-226	*Xylaria ellisii*	New Brunswick, CA	Blueberry
E-227	*Hypoderma* cf. *rubi*	New Brunswick, CA	Blueberry
E-228	*Paraphaeosphaeria neglecta*	Debert, NS	Blueberry
E-229	*Diaporthe vaccinii*	Portapique, NS	Blueberry

**Endophyte ID**	**Tentative species ID**	**Origin**	**Host Organism**
E-231	Tympanidaceae sp.	Mt. Thom, NS	Blueberry
E-232	*Epicoleosporium ramularioides*	Mt. Thom, NS	Blueberry
E-233	*Seimatosporium lichenicola*	New Brunswick, CA	Blueberry
E-234	PCR unsuccessful	New Brunswick, CA	Blueberry
E-235	*Seimatosporium lichenicola*	Portapique, NS	Blueberry
E-236	*Creosphaeria sassafras*	Jordan Station, ON	Grape
E-237	*Colletotrichum* cf. *truncatum*	Mt. Thom, NS	Blueberry
E-238	*Hypoderma* cf. *rubi*	New Brunswick, CA	Blueberry
E-239	*Hypoderma* cf. *rubi*	New Brunswick, CA	Blueberry
E-240	Tympanidaceae sp.	New Brunswick, CA	Blueberry
E-241	*Xylaria ellisii*	New Brunswick, CA	Blueberry
E-242	PCR unsuccessful	New Brunswick, CA	Blueberry
E-243	*Xylaria ellisii*	Mt. Thom, NS	Blueberry
E-244	*Xylaria ellisii*	Mt. Thom, NS	Blueberry
E-245	*Godronia cassandrae*	Mt. Thom, NS	Blueberry
E-246	*Godronia cassandrae*	Mt. Thom, NS	Blueberry
E-247	Tympanidaceae sp.	Mt. Thom, NS	Blueberry
E-248	*Ramularia* sp.	New Brunswick, CA	Blueberry
E-249	Xylariaceae sp.	Jordan Station, ON	Blueberry
E-250	*Hypomontagnella submonticulosa*	Jordan Station, ON	Grape
E-251	*Hypomontagnella submonticulosa*	Jordan Station, ON	Raspberry
E-252	*Ramularia* sp.	New Brunswick, CA	Blueberry
E-253	*Ramularia* sp.	New Brunswick, CA	Blueberry
E-254	PCR unsuccessful	New Brunswick, CA	Blueberry
E-255	Tympanidaceae sp.	New Brunswick, CA	Blueberry
E-256	*Phyllosticta* cf. *pyrolae*	Mt. Thom, NS	Blueberry
E-257	*Ramularia* sp.	New Brunswick, CA	Blueberry
E-258	*Godronia cassandrae*	New Brunswick, CA	Blueberry
E-259	*Fusarium tricinctum*	New Brunswick, CA	Blueberry
E-260	*Sphaerulina vaccinii*	Portapique, NS	Blueberry
E-261	*Nigrospora sphaerica*	Simcoe, ON	Raspberry

Endophyte ID	Tentative species ID	Origin	Host Organism
E-262	*Colletotrichum fioriniae*	New Brunswick, CA	Blueberry
E-263	*Paraphaeosphaeria neglecta*	Mt. Thom, NS	Blueberry
E-300	*Aposphaeria corallinolutea*	AAFC Kentville, NS	Blueberry
E-301	*Nemania serpens*	AAFC Kentville, NS	Blueberry
E-303	*Xylaria ellisii*	AAFC Kentville, NS	Blueberry
E-304	*Neocucurbitaria* sp.	AAFC Kentville, NS	Blueberry
E-305	*Neocucurbitaria* sp.	AAFC Kentville, NS	Blueberry
E-306	*Pseudoplectania nigrella*	AAFC Kentville, NS	Blueberry
E-307	PCR unsuccessful	AAFC Kentville, NS	Blueberry
E-308	*Aspergillus amstelodami*	AAFC Kentville, NS	Blueberry
E-309	PCR unsuccessful	AAFC Kentville, NS	Blueberry
E-310	*Cladosporium* cf. *cladosporioides*	AAFC Kentville, NS	Blueberry
E-312	*Neocucurbitaria* sp.	AAFC Kentville, NS	Blueberry
E-313	*Neocucurbitaria* sp.	AAFC Kentville, NS	Blueberry
E-314	*Neocucurbitaria* sp.	AAFC Kentville, NS	Blueberry
E-316	*Pseudoplectania nigrella*	AAFC Kentville, NS	Blueberry
E-317	*Xylaria ellisii*	AAFC Kentville, NS	Blueberry
E-319	*Paraphaeosphaeria neglecta*	Debert, NS	Lowbush blueberry
E-320	*Paraphaeosphaeria neglecta*	Debert, NS	Lowbush blueberry
E-321	*Neocucurbitaria* sp.	Debert, NS	Lowbush blueberry
E-322	*Paraphaeosphaeria neglecta*	Debert, NS	Lowbush blueberry
E-323	*Neocucurbitaria* sp.	AAFC Kentville, NS	Blueberry
E-324	*Proliferodiscus* sp.	AAFC Kentville, NS	Blueberry
E-326	*Proliferodiscus* sp.	AAFC Kentville, NS	Blueberry
E-327	*Penicillium camponotum*	AAFC Kentville, NS	Blueberry
E-330	*Proliferodiscus* sp.	AAFC Kentville, NS	Blueberry
E-331	*Proliferodiscus* sp.	AAFC Kentville, NS	Blueberry
E-332	*Proliferodiscus* sp.	AAFC Kentville, NS	Blueberry
E-333	*Neocucurbitaria* sp.	AAFC Kentville, NS	Blueberry
E-334	*Gloeophyllum sepiarium*	Mt. Thom, NS	Lowbush blueberry
E-335	*Cladosporium allicinum*	Debert, NS	Lowbush blueberry
E-336	*Plagiostoma petiolophilum*	Mt. Thom, NS	Lowbush blueberry
E-337	*Cytospora ribis*	Debert, NS	Lowbush blueberry

Endophyte ID	Tentative species ID	Origin	Host Organism
E-338	*Thyronectria cucurbitula*	Kemptown, NS	Lowbush blueberry
E-339	*Neocucurbitaria* sp.	Debert, NS	Lowbush blueberry
E-340	*Leptodontidium* sp.	Debert, NS	Lowbush blueberry
E-341	*Leptodontidium* sp.	Debert, NS	Lowbush blueberry
E-342	*Trichaptum abietinum*	Kemptown, NS	Lowbush blueberry
E-343	*Lachnellula calyciformis*	Kemptown, NS	Lowbush blueberry
E-344	*Pseudoplectania nigrella*	Debert, NS	Lowbush blueberry
E-346	*Hypomontagnella submonticulosa*	AAFC Kentville, NS	Blueberry
E-347	*Xylaria ellisii*	AAFC Kentville, NS	Blueberry
E-349	*Trichaptum abietinum*	AAFC Kentville, NS	Blueberry
E-350	*Lachnellula calyciformis*	Kemptown, NS	Lowbush blueberry
E-351	*Sarea difformis*	Mt. Thom, NS	Lowbush blueberry
E-352	*Seimatosporium lichenicola*	AAFC Kentville, NS	Blueberry
E-353	*Proliferodiscus* sp.	AAFC Kentville, NS	Blueberry
E-354	*Xylaria ellisii*	AAFC Kentville, NS	Blueberry
E-356	*Neocucurbitaria* sp.	AAFC Kentville, NS	Blueberry
E-357	*Seimatosporium lichenicola*	AAFC Kentville, NS	Blueberry
E-360	*Botrytis cinerea*	Mt. Thom, NS	Lowbush blueberry
E-361	*Proliferodiscus* sp.	AAFC Kentville, NS	Blueberry
E-362	*Aposphaeria corallinolutea*	AAFC Kentville, NS	Blueberry
E-363	*Proliferodiscus* sp.	AAFC Kentville, NS	Blueberry
E-366	*Neocucurbitaria* sp.	AAFC Kentville, NS	Blueberry
E-367	*Nemania* sp.	AAFC Kentville, NS	Blueberry
E-368	*Cytospora* cf. *cedri*	AAFC Kentville, NS	Blueberry
E-369	*Neocucurbitaria* sp.	AAFC Kentville, NS	Blueberry
E-370	*Alternaria* cf. *alternata*	AAFC Kentville, NS	Blueberry
E-372	*Seimatosporium lichenicola*	AAFC Kentville, NS	Blueberry
E-373	*Neocucurbitaria* sp.	Debert, NS	Lowbush blueberry
E-375	*Xylaria ellisii*	AAFC Kentville, NS	Blueberry

Table S2. Natural products dereplicated from molecular network of Canadian fungal endophytes. Formulas listed do not include adducts.

Cluster	Compound	Measured *m/z*	Formula	RT (min)	Mass error (Δppm)	Major producers
A	Hormonemate F	626.3747 $[M+NH_4]^+$	$C_{29}H_{52}O_{13}$	4.36	0.116	Dothideaceae sp.
	Hormonemate A/E	740.4426 $[M+NH_4]^+$	$C_{35}H_{62}O_{15}$	4.75	-0.171	Dothideaceae sp.
	Hormonemate[a]	770.4531 $[M+NH_4]^+$	$C_{36}H_{64}O_{16}$	4.64	-0.170	Dothideaceae sp.
	[3-methyl-1-[3-methyl-1-[3-methyl-1-[3-methyl-1-[3-methyl-1-oxo-1-(2,3,4,5,6-pentahydroxyhexoxy)pentan-2-yl]oxy-1-oxopentan-2-yl]oxy-1-oxopentan-2-yl]oxy-1-oxopentan-2-yl] 2-acetyloxy-3-methylpentanoate[a]	812.4634 $[M+NH_4]^+$	$C_{38}H_{66}O_{17}$	4.83	-0.711	Dothideaceae sp.
B	Phenochalasin C	450.2639 $[M+H]^+$	$C_{28}H_{35}NO_4$	4.15	-0.145	*Xylaria ellisii*
	Cytochalasin Z26/Z22/Z23	480.2382 $[M+H]^+$	$C_{28}H_{33}NO_6$	3.72	0.199	*Xylaria ellisii*
	Zygosporin E	492.2744 $[M+H]^+$	$C_{30}H_{37}NO_5$	4.37	0.244	*Xylaria ellisii*
	Epoxycytochalasin C/D	524.2645 $[M+H]^+$	$C_{30}H_{37}NO_7$	3.53	0.479	*Xylaria ellisii*
	Epoxycytochalasin N/R	540.2593 $[M+H]^+$	$C_{30}H_{37}NO_8$	3.75	0.160	*Xylaria ellisii*
	Cytochalasin P1	542.2749 $[M+H]^+$	$C_{30}H_{39}NO_8$	3.54	-0.007	*Xylaria ellisii*
C	Enniatin B3/J1	629.4120 $[M+NH_4]^+$	$C_{31}H_{53}N_3O_9$	4.84	0.070	*Fusarium tricinctum*
	Enniatin B2/J2/K	643.4276 $[M+NH_4]^+$	$C_{32}H_{55}N_3O_9$	4.91	0.224	*Fusarium tricinctum*
	Enniatin B	657.4443 $[M+NH_4]^+$	$C_{33}H_{57}N_3O_9$	4.95	1.010	*Fusarium tricinctum*

Cluster	Compound	Measured *m/z*	Formula	RT (min)	Mass error (Δppm)	Major producers
	Enniatin P1	659.4223 $[M+NH_4]^+$	$C_{32}H_{55}N_3O_{10}$	4.55	-0.410	*Fusarium tricinctum*
	Enniatin P2	673.4382 $[M+NH_4]^+$	$C_{33}H_{57}N_3O_{10}$	4.68	-0.075	*Fusarium tricinctum*
	Enniatin A1/E1/E2/G/O2	685.4746 $[M+NH_4]^+$	$C_{35}H_{61}N_3O_9$	5.24	0.269	*Fusarium tricinctum*
	Enniatin A/A2/C/F/MK1688	699.4902 $[M+NH_4]^+$	$C_{36}H_{63}N_3O_9$	5.32	-0.209	*Fusarium tricinctum*
	Enniatin M1/M2	701.4698 $[M+NH_4]^+$	$C_{35}H_{61}N_3O_{10}$	4.86	0.470	*Fusarium tricinctum*
D	Lovastatin[a]	405.2631 $[M+H]^+$	$C_{24}H_{36}O_5$	4.50	-1.211	*Seimatosporium lichenicola*
	Simvastatin[a]	419.2793 $[M+H]^+$	$C_{25}H_{38}O_5$	4.74	-0.073	*Seimatosporium lichenicola*
	Lovastatin analogue[a]	482.3110 $[M+NH_4]^+$	$C_{26}H_{40}O_7$	4.07	-0.392	*Seimatosporium lichenicola*
E	Ellisiiamide D	522.3649 $[M+H]^+$	$C_{27}H_{47}N_5O_5$	4.28	-0.012	*Xylaria ellisii*
	Cyclic pentapeptide 2	536.3804 $[M+H]^+$	$C_{28}H_{49}N_5O_5$	4.36	-0.515	*Xylaria ellisii*
	Xylarotide A	550.3968 $[M+H]^+$	$C_{29}H_{51}N_5O_5$	4.51	0.225	*Xylaria ellisii*
	Ellisiiamide A	556.3494 $[M+H]^+$	$C_{30}H_{45}N_5O_5$	4.65	0.043	*Xylaria ellisii*
	Ellisiiamide B	570.3649 $[M+H]^+$	$C_{31}H_{47}N_5O_5$	4.71	-0.116	*Xylaria ellisii*
	Cyclic pentapeptide 1	584.3807 $[M+H]^+$	$C_{32}H_{49}N_5O_5$	4.66	0.161	*Xylaria ellisii*
	Ellisiiamide C	598.3961 $[M+H]^+$	$C_{33}H_{51}N_5O_5$	4.76	-0.294	*Xylaria ellisii*
	Ellisiiamide G	600.3754 $[M+H]^+$	$C_{32}H_{49}N_5O_6$	4.14	-0.218	*Xylaria ellisii*
F	Demethylsambutoxin	440.2793 $[M+H]^+$	$C_{27}H_{37}NO_4$	4.85	-0.534	*Fusarium tricinctum*
	Sambutoxin	454.2952 $[M+H]^+$	$C_{28}H_{39}NO_4$	5.08	-0.033	*Fusarium tricinctum*
	Anhydrooxysporidinone	472.3059 $[M+H]^+$	$C_{28}H_{41}NO_5$	4.81	0.403	*Fusarium tricinctum*
	Oxysporidinone[a]	490.3162 $[M+H]^+$	$C_{28}H_{43}NO_6$	4.73	-0.132	*Fusarium tricinctum*

Cluster	Compound	Measured *m/z*	Formula	RT (min)	Mass error (Δppm)	Major producers
G	Trypethelone	273.1120 $[M+H]^+$	$C_{16}H_{16}O_4$	4.05	-0.496	*Godronia cassandrae*
	Sclerodin	329.1019 $[M+H]^+$	$C_{18}H_{16}O_6$	4.51	-0.288	*Godronia cassandrae*
	Sclerodinol	345.0969 $[M+H]^+$	$C_{18}H_{16}O_7$	4.30	0.292	*Godronia cassandrae*
H	[3-methyl-1-[3-methyl-1-[3-methyl-1-[3-methyl-1-oxo-1-(2,3,4,5-tetrahydroxypentoxy)pentan-2-yl]oxy-1-oxopentan-2-yl]oxy-1-oxopentan-2-yl]oxy-1-oxopentan-2-yl] 2-acetyloxy-3-methylpentanoate[a]	782.4527 $[M+NH_4]^+$	$C_{37}H_{64}O_{16}$	5.03	-0.641	Dothideaceae sp.
I	Coleophomone intermediate	469.1857 $[M+H]^+$	$C_{26}H_{28}O_8$	3.79	0.076	*Xylaria cubensis*
J	(E)-4-(6,8-dihydroxy-3-methyl-1-oxoisochromen-7-yl)-2-methoxybut-3-enoic acid[a]	307.0812 $[M+H]^+$	$C_{15}H_{14}O_7$	3.52	-0.258	*Ramularia, Epicoccum, and Mollisia* spp.
K	Cytochalasin D	508.2696 $[M+H]^+$	$C_{30}H_{37}NO_6$	4.05	-0.427	*Xylaria ellisii*
L	Griseophenone C	305.1019 $[M+H]^+$	$C_{16}H_{16}O_6$	3.41	-0.212	*Xylaria ellisii*
	Dechlorogriseofulvin[a]	319.1179 $[M+H]^+$	$C_{17}H_{18}O_6$	4.06	1.144	*Xylaria ellisii*
	Griseofulvin[a]	353.0786 $[M+H]^+$	$C_{17}H_{17}ClO_6$	4.01	0.078	*Xylaria ellisii*
M	(E)-5-(4-methoxy-5-methyl-6-oxopyran-2-yl)-	267.1226 $[M+H]^+$	$C_{14}H_{18}O_5$	3.57	-0.300	*Seimatosporium lichenicola*

Cluster	Compound	Measured *m/z*	Formula	RT (min)	Mass error (Δppm)	Major producers
	3-methylhex-4-enoic acid[a] (similar to marinopyrones)					
N	Senkyunolide H[a]	225.1122 $[M+H]^+$	$C_{12}H_{16}O_4$	3.60	0.153	*Nigrospora sphaerica* and *Cytospora ribis*
O	Trichodin A analogue[a]	354.2063 $[M+H]^+$	$C_{22}H_{27}NO_3$	3.86	-0.452	*Cryptosporella femoralis*
P	Hirsutatin A	677.3756 $[M+H]^+$	$C_{34}H_{52}N_4O_{10}$	4.53	-0.104	*Xylaria ellisii*
Q	Trienylfuranone A/B	179.1067 $[M+H]^+$	$C_{11}H_{14}O_2$	2.93	-0.091	*Hypomontagnella submonticulosa*
	Trienylfuranol A	181.1223 $[M+H]^+$	$C_{11}H_{16}O_2$	3.73	0.131	*Hypomontagnella submonticulosa*
R	LL P880 beta (fungal lactone)[a]	231.1228 $[M+H]^+$	$C_{11}H_{18}O_5$	3.12	0.389	*Xylaria ellisii*
S	Deoxylachnellulone	309.2059 $[M+H]^+$	$C_{18}H_{28}O_4$	4.58	-0.342	*Lachnellula calyciformis*
	Lachnellulone	325.2009 $[M+H]^+$	$C_{18}H_{28}O_5$	4.22	-0.063	*Lachnellula calyciformis*

[a]Dereplicated with GNPS Library search function
